# Unveiling Correlations in Metal‐Organic Interface Properties: A Computational Exploration of Alternant and Non‐Alternant π‐Electron Systems

**DOI:** 10.1002/cplu.202400771

**Published:** 2025-03-20

**Authors:** Jakob Schramm, Ralf Tonner‐Zech

**Affiliations:** ^1^ Wilhelm-Ostwald-Institut für Physikalische und Theoretische Chemie Leipzig University Linnéstr. 2 04103 Leipzig Germany

**Keywords:** Chemisorption, Physisorption, Interfaces, Polycycles, Density functional calculations

## Abstract

Metal‐organic interfaces are critical in organic electronic devices, influencing key performance properties. Understanding these relationships is essential for improving such devices. Polycyclic conjugated hydrocarbons (PCHs) with alternant and non‐alternant topologies are promising candidates for exploring these interfaces since they show physisorption and chemisorption, respectively. Using density functional theory with periodic boundary conditions, we modeled the interfaces between a Cu(111) surface and 22 PCHs (11 alternant and 11 non‐alternant). We identified quantitative correlations among interface properties, showing that these properties form a “fixed set” of properties for individual molecules. A clear distinction emerges between physisorption and chemisorption for most properties, except for work function changes, which are consistently governed by the Pauli pushback effect resulting from dispersion pull. Interestingly, molecules with larger π‐electron systems exhibit stronger dispersion attraction yet higher adsorption heights. This study provides chemically intuitive explanations for these findings and highlights the interconnected nature of interface properties. The insights gained offer valuable guidance for understanding and optimizing Cu(111)‐organic interfaces, contributing to advancements in organic electronics.

## Graphical abstract

The properties of metal‐organic interfaces are important for the performance of organic electronic devices. The adsorption of alternant and non‐alternant polycyclic conjugated hydrocarbons on Cu(111) was modeled using first‐principle DFT calculations. The difference in interface properties between physisorption and chemisorption is shown and relations between interface properties are identified.

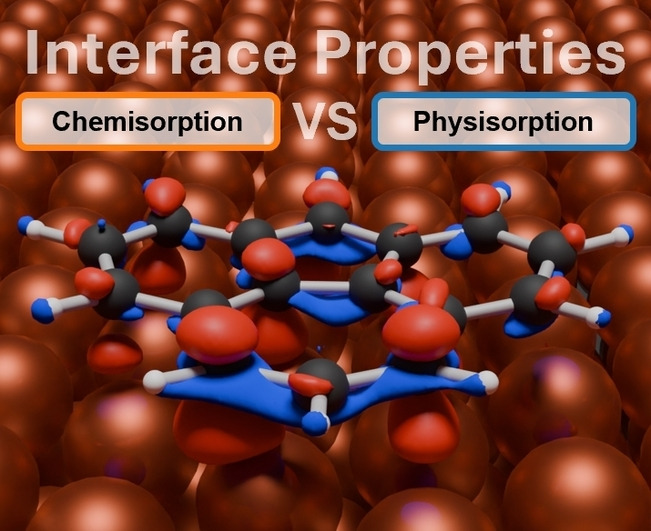

## 1 Introduction

Carbon‐based materials with polycyclic π‐electron systems are widely used as organic semiconductors in (opto‐)electronic devices like organic field‐effect transistors (OFETs), organic light‐emitting diodes (OLEDs), or organic solar cells (OSCs).[^1^, ^2^, ^3^, ^4^] Simple molecules like polycyclic aromatic hydrocarbons (PAHs), or more generally polycyclic conjugated hydrocarbons (PCHs), polymers and graphene derivatives appear to be promising candidates for applications.[^4^, ^5^, ^6^, ^7^, ^8^, ^9^, ^10^, ^11^]

In such devices, the organic semiconductor is typically contacted by a conducting metal electrode, forming a so‐called metal‐organic interface.[^12^, ^13^, ^14^] This interface is crucial for the performance of the device since it determines important parameters like charge carrier injection rates.[^12^, ^13^, ^14^] Therefore, understanding such metal‐organic interfaces is necessary for improvement of organic electronic devices. In surface science, this is usually accomplished by investigating model systems.^[15]^

Interesting systems that have been experimentally investigated in recent years are isomer pairs of non‐alternant and alternant PCHs on transition metal (111) surfaces.[^16^, ^17^, ^18^, ^19^, ^20^, ^21^] The pairs differ in the topology of the carbon backbone: While for alternant molecules it is possible to label all carbon atoms alternatingly (e. g. with red and green labels) such that neighboring carbon atoms have different labels, this is not possible for non‐alternant ones.^[22]^ The latter is due to the presence of odd‐numbered, usually 5‐ or 7‐membered rings. The difference in topology leads to a changed electronic structure, which can also lead to a difference in interaction with the substrate. While on Pt(111) and Ag(111), the isomer pairs show a rather similar behavior,[^17^, ^18^, ^19^] the difference between them is especially present on the Cu(111) surface: Alternant molecules like naphthalene or pyrene adsorb in a flat configuration, show no stabilizing electronic interactions as well as no significant charge transfer and are only bound by dispersion forces.[^16^, ^17^, ^18^, ^20^, ^21^] These observations lead to the conclusion that they are physisorbed on the Cu(111) surface. In contrast, the respective non‐alternant isomers like azulene, azupyrene and acepleiadylene have a significant lower adsorption height, are bent and have a significantly stabilizing electronic interaction with a surface‐to‐molecule charge transfer, which means they can be classified as chemisorbed on the Cu(111) surface.[^16^, ^17^, ^18^, ^20^, ^21^]

Although those experimental and theoretical studies were an important first step for understanding the metal‐organic interfaces, a bigger picture of the interface properties in both adsorption regimes, physisorption and chemisorption, as well as if and how they are connected to each other is missing. This is only possible by investigating a surface that shows both adsorption regimes and by utilizing a diverse set of molecules that covers both adsorption regimes as well as different strengths within one category. Thus, a combined set of non‐alternant and alternant molecules adsorbing on the Cu(111) surface is promising here.

**Scheme 1:**
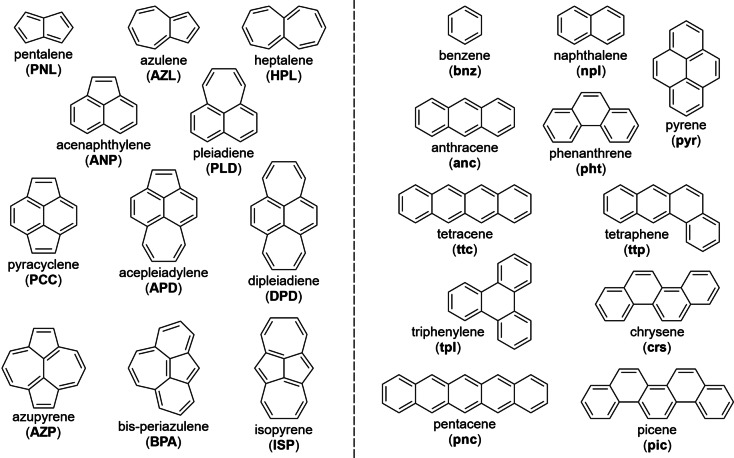
Investigated non‐alternant (left, capitalized abbreviations) and alternant π‐electron systems (right, lower case abbreviations) shown with one possible Kekulé structure.

Despite recent progress,[^6^, ^23^, ^24^, ^25^, ^26^] the synthesis of non‐alternant molecules with subsequent adsorption studies remains often challenging. Therefore, we used first‐principles *in‐silico* screening of a set of 11 non‐alternant PCHs as well as 11 corresponding alternant PCHs for comparison (see Scheme 1) for the adsorption on the Cu(111) surface and the resulting interface properties. This not only makes it less time‐consuming and less resource‐intensive, but also enables the investigation of molecules that are highly unstable (e. g., **PNL** ^[27]^) or have not yet been synthesized (e. g., **BPA**[^28^, ^29^]). Based on previous combined experimental and theoretical studies on prototypical systems,[^16^, ^17^, ^18^, ^19^, ^20^, ^21^, ^30^] we conclude that the methodology used herein has predictive nature.

Both sets of molecules were selected with a systematic approach: The non‐alternant molecules were limited to 5‐, 6‐ and 7‐membered rings and must possess a possible Kekulé structure, i. e., a Lewis structure with single and double bonds without unpaired electrons. This leads to the following set: all possible bicyclic molecules (**PNL**, **AZL**, and **HPL**), all possible tricyclic naphthalene derivatives (**ANP** and **PLD**), all possible quadricyclic naphthalene derivatives annulated in the bay region (**PCC**, **APD**, and **DPD**), and derivatives of the bicyclic molecules annulated with two corresponding rings in the bay region (**HPL**+5, **AZL**+6, and **PNL**+7) to yield a pyrene isomer (**AZP**, **BPA**, and **ISP**).

The alternant molecules were limited to 6‐membered rings (benzenoid), must possess a possible Kekulé structure, need to be planar and should be in a similar size range as the non‐alternant ones. The following were derived from this: all monocyclic molecules (**bnz**), all bicyclic molecules (**npl**), all tricyclic molecules (**anc** and **pht**), all (planar) quadricyclic molecules (**pyr**, **ttc**, **ttp**, **tpl**, and **crs**), and pentacyclic (zigzag) acene (**pnc**) and (armchair) phenacene (**pic**).

We now use density functional theory (DFT) calculations employing a slab ansatz to compute interface properties and investigate quantitative correlations between them. In this way, we will gain a better understanding of the mechanisms of how the properties of Cu(111)‐organic interfaces are formed and what the physicochemical relations between them are.

## 2 Theoretical Background

This section introduces the different interface properties used for the analysis. The exact definitions of all quantities can be found in Supporting Information Section 1. They characterize different aspects of the Cu(111)‐organic interface, some of which are even experimentally accessible. They can be divided into three different groups:

- energetic aspect,
- geometric aspect, and
- electronic aspect.

All interface properties were determined by DFT calculations employing a slab ansatz within periodic boundary conditions (for more details see section 3).

### 2.1 (A) Energetic Aspect of the Interface

**Scheme 2:**
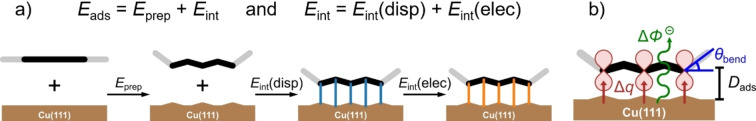
Schematic representation of the investigated interface properties. a) Steps of the energy decomposition of the adsorption energy and b) geometric and electronic interface properties. See text for discussion.

The main quantity used for analysis of energetic interface properties is the adsorption energy (*E*_ads_). However, it can be very useful to decompose it into meaningful contributions. First, it can be decomposed into a preparation energy (*E*_prep_), i. e. the energy needed for deformation of the molecule [*E*_prep_(mol)] and the surface [*E*_prep_(surf)], and an interaction energy (*E*_int_), i. e. the bonding energy between the distorted fragments. Furthermore, since the additive DFT−D3 dispersion correction[^31^, ^32^] was used, *E*_int_ can be decomposed into a dispersion interaction energy [*E*_int_(disp)] based on the D3 energies and an electronic interaction energy [*E*_int_(elec)] based on the Kohn‐Sham energies. This decomposition and the corresponding equations are schematically shown in Scheme 2a and summarized in Table 1 (for more details see Supporting Information Section 1.1). *E*_int_(elec) is particularly important, as its sign determines how we classify an adsorption process: If it is positive and therefore destabilizing, it is called physisorption (phys), or if it is negative and therefore stabilizing, it is called chemisorption (chem).^[18]^

**Table 1:** Overview of the interface properties investigated, grouped into (A) energetic, (B) geometric, and (c) electronic aspects.

Interface Properties		
(A)	adsorption energy	*E*_ads_
	preparation energy	*E*_prep_
	interaction energy	*E*_int_
	dispersion interaction energy	*E*_int_(disp)
	electronic interaction energy	*E*_int_(elec)
(B)	adsorption height	*D*_ads_
	molecular corrugation	*D*_ads_^MAD^
	bending angle	θ_bend_
(C)	charge transfer	Δ*q*
	change of work function	ΔΦ

### 2.2 (B) Geometric Aspect of the Interface

The structure of the interface is quantitatively described by the adsorption height (*D*_ads_), calculated as the mean of the C atoms relative to a relaxed surface. Additionally, the mean absolute deviation (MAD) of the C atoms relative to a relaxed surface was calculated to quantify the corrugation of the molecule (*D*_ads_^MAD^). The structure of the adsorbed molecule can be furthermore characterized by the bending angle of the C−H bonds out of the molecular plane (θ_bend_). The geometric interface properties are visualized in Scheme 2b and summarized in Table 1 (for more details see Supporting Information Section 1.2).

### 2.3 (C) Electronic Aspect of the Interface

The electronic change of the pristine surface due to the formation of an interface by adsorption of a molecule was characterized by two quantities: The charge transfer (Δ*q*) based on the Hirshfeld scheme[^20^, ^33^] as well as the change of work function (ΔΦ) of the Cu(111) surface were investigated. The electronic interface properties are schematically shown in Scheme 2b and summarized in Table 1 (for more details see Supporting Information Section 1.3).

## 3 Computational Methods

The adsorption of the investigated molecules on the Cu(111) surface was modeled employing a slab ansatz and using DFT with periodic boundary conditions implemented in the Vienna Ab Initio Simulation Package (VASP) version 5.4.4.[^34^, ^35^, ^36^, ^37^] The generalized gradient approximation (GGA) based density functional proposed by Perdew, Burke and Ernzerhof (PBE)[^38^, ^39^] was used together with the third‐generation van der Waals dispersion correction by Grimme et al. (DFT−D3)^[31]^ with Becke‐Johnson (BJ)^[32]^ damping as it previously showed good agreement with experimental results for such systems.[^16^, ^18^, ^20^, ^21^] As the basis set, plane‐waves within the projector‐augmented wave (PAW) ansatz were used.[^40^, ^41^]

The previously determined lattice parameter for copper with 3.568 Å^[16]^ was employed to construct a 4‐layer slab of the (111) surface. To avoid interactions of an adsorbed molecule with its periodic image, an 8x8 super cell was used, which leads to structures close to the single‐molecule limit. During optimizations, the bottom two layers were kept frozen at their bulk positions to preserve this bulk structure at the bottom, while the top two layers and the molecule were optimized.

The plane‐wave cutoff energy, the vacuum layer thickness, and the k‐mesh were also previously determined by convergence series^[16]^ yielding a cutoff energy of 350 eV, a vacuum layer of 30 Å, and a Γ‐centered 24×24×1 Monkhorst‐Pack k‐point mesh for the unit cell, which was adjusted to the 8×8 supercell yielding a 3×3×1 k‐point mesh. The 1^st^ order Methfessel‐Paxton smearing method with a smearing width of 0.2 eV was employed to improve self‐consistent field (SCF) convergence. The SCF convergence criterion was set to 10^−5^ eV and all structures were optimized until changes in forces were below 10^−2^ eV/Å.

An extensive and systematic search for the best adsorption site was carried out. First, for all non‐alternant molecules, 30 single point calculations at an adsorption height of 2.5 Å were carried out. There, one random central quaternary carbon atom of the molecule was set at the on‐top, at both hollow (hcp and fcc), as well as at all three bridge positions of the unit cell and rotations of the whole molecule of 0°, 15°, 30°, 45°, and 60° were considered for each position (higher rotation angles lead to symmetry‐equivalent structures). Next, up to 5 of the energetically most stable structures (omitting symmetry‐equivalent ones) were taken and structurally optimized. The structure with the lowest energy after the optimization was then used for further analysis. For alternant molecules, the procedure was kept simpler. Since the 6‐membered benzenoid rings fit very well on the hexagonal lattice of the Cu(111) surface, only structures with the carbon atoms at on‐top and hollow sites were optimized. There, always the structures where the carbon atoms are at fcc hollow sites and the rings are above hcp hollow sites yielded the lowest energy in agreement to literature[^16^, ^20^, ^21^, ^42^, ^43^, ^44^, ^45^, ^46^] and were used for further analysis. Top views on all final adsorption structures obtained can be found in the Supporting Information Figures S7 and S8.

Atomic partial charge analysis was carried out using the Hirshfeld scheme.^[33]^ This was achieved in a separate single point calculation using IVDW=20, as the Tkatchenko‐Scheffler dispersion correction^[47]^ requires a Hirshfeld analysis of the electron density calculated by VASP. Note that the different dispersion correction has no influence on the electronic structure of the system. The vacuum potential for analysis of the work function Φ was determined from the average in xy‐plane of the Hartree potential in the vacuum region, where the change of the slope was constant. These calculations were carried out using the dipole correction.[^48^, ^49^]

All data used for further analysis can be found in the Supporting Information Tables S1, S2, and S3.

## 4 Results and Discussion

Our goal is to see how adsorption in the two regimes, chemisorption and physisorption, will affect the interface properties in comparison. Furthermore, we want to understand how the interface properties within an adsorption regime are quantitatively connected to each other. Therefore, the different interface properties were plotted against each other. In this way, the size range in which the respective regime lies can be recognized. Additionally, linear correlations can be easily visualized and evaluated based on the coefficient of determination (R^2^). After identifying correlations (Supporting Information Figure S1), we will rationalize them on a chemically intuitive basis. In this way, we can fully understand the formation of the Cu(111)‐organic interface with its corresponding properties for different adsorption regimes and interaction strengths.

First, we need to identify which of the molecules will chemisorb and which will physisorb. Afterwards, we will discuss the different aspects of the Cu(111)‐organic interface starting with the energetic, followed by the geometric and finalized with the electronic aspect.

### 4.1 (A) Energetic Aspect of the Metal‐Organic Interface

According to IUPAC, chemisorption is an adsorption which results from chemical bond formation, while physisorption is an adsorption in which van der Waals forces are involved and which do not involve a significant change in the electronic orbital patterns of the species involved.^[50]^ To have a simple quantitative measure for the distinction, we use the electronic interaction energy [*E*_int_(elec)] obtained from the decomposition of the adsorption energy (*E*_ads_; Scheme 2a) and call a molecule chemisorbed if *E*_int_(elec) is negative, while we will call it physisorbed if *E*_int_(elec) is positive. In previous combined experimental and theoretical work, it was shown that a negative *E*_int_(elec) corresponds to the formation of a covalent bond, while a positive *E*_int_(elec) corresponds to a dispersion interaction dominated bond, which is not a chemical bond in most common definitions.[^16^, ^17^, ^18^, ^20^] Since the molecules of the previous work belong to the same substance class and are also part of the molecules investigated here, we assume that a negative *E*_int_(elec) describes a very similar formation of a chemical bond without an explicit confirmation by a quantitative bonding analysis. Of course, using the latter would reveal additional information about the chemical bond between the surface and the molecule. But as the nature of the chemical bond is not relevant for classification – it is just about whether there is one – we did not quantify chemical bonding. Furthermore, as it will become apparent later, using the sign of *E*_int_(elec) is a reliable method for classifying chemisorption and physisorption, because the classified molecules show significant differences in most interface properties as expected. However, it should be noted that the results, including the classification, are only as good as the description of the metal‐organic interface by the dispersion corrected DFT method used.

#### 4.1.1 Classification of Adsorption Behavior from Electronic Interaction

**Figure 1:**
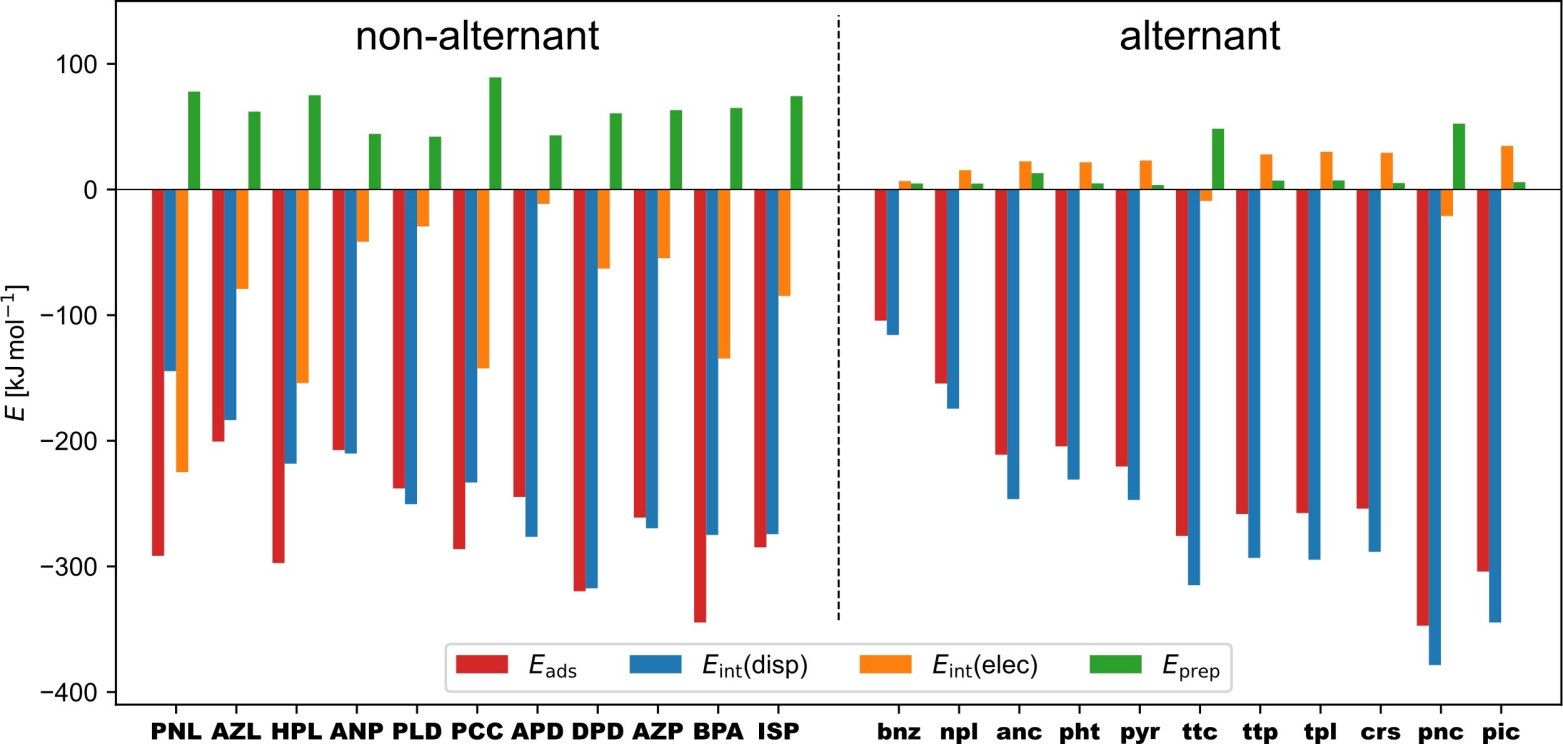
Adsorption energy *E*_ads_ and its decomposed contributions dispersion interaction energy *E*_int_(disp), electronic interaction energy *E*_int_(elec), and preparation energy *E*_prep_ of all investigated non‐alternant (left) and alternant π‐electron systems (right), with the corresponding acronyms being defined in Scheme 1.

All non‐alternant molecules show a negative and therefore stabilizing *E*_int_(elec) (−11 to −225 kJ/mol; Figure 1, left) demonstrating the formation of a chemical bond to the Cu(111) surface and thus chemisorption. In addition, there are two alternant molecules, **ttc** and **pnc**, which also show a negative *E*_int_(elec) (−9 and −21 kJ/mol; Figure 1, right). Consequently, these two are also considered to be chemisorbed on the Cu(111) surface. Note that *E*_int_(elec) ranges from a few kJ/mol up to several hundred, showing that there is a wide range from weak to strong chemisorption. All other alternant molecules show a positive and therefore destabilizing *E*_int_(elec) (+7 to +35 kJ/mol; Figure 1, right), which means they are solely bound by dispersion interactions and thus physisorbed.

This implies that the topology of the carbon backbone has an influence on the adsorption regime. However, it is not only the classification into non‐alternant or alternant topology that is decisive, as two alternant molecules are also chemisorbed. These two molecules belong to the acene family and are the two longest ones of the five acenes investigated here.[^5^, ^51^] Acenes are known to become more reactive the longer they become: Qualitatively, only one Clar sextet, i. e. a benzene unit of alternating single and double bonds, can be formulated, regardless of the length of the acene. This reduces the stability and increases the reactivity with increasing acene length.[^5^, ^51^] Thus, this is also an effect of the molecule's topology. To investigate the different adsorption regimes independently, the set of molecules is divided into chemisorbed (chem) and physisorbed (phys) molecules for all further considerations.

#### 4.1.2 Influence of Dispersion Interaction

Although we used the sign of *E*_int_(elec) to determine the adsorption behavior, further discussion of the dispersion interaction energy [*E*_int_(disp)] is necessary for a detailed understanding of the energetic situation at the Cu(111)‐organic interface.

Note that for most molecules we classified as chemisorbed, the stabilizing total interaction energy (*E*_int_) is still dominated by *E*_int_(disp) as the ratio *E*_int_(disp)/*E*_int_ is larger than 50 % (59 to 97 %; except for **PNL** with 39 %; Supporting Information Table S2). Nevertheless, we will show in the next sections that even a small electronic contribution shifts the interface properties into a different regime compared to physisorbed molecules.

**Figure 2:**
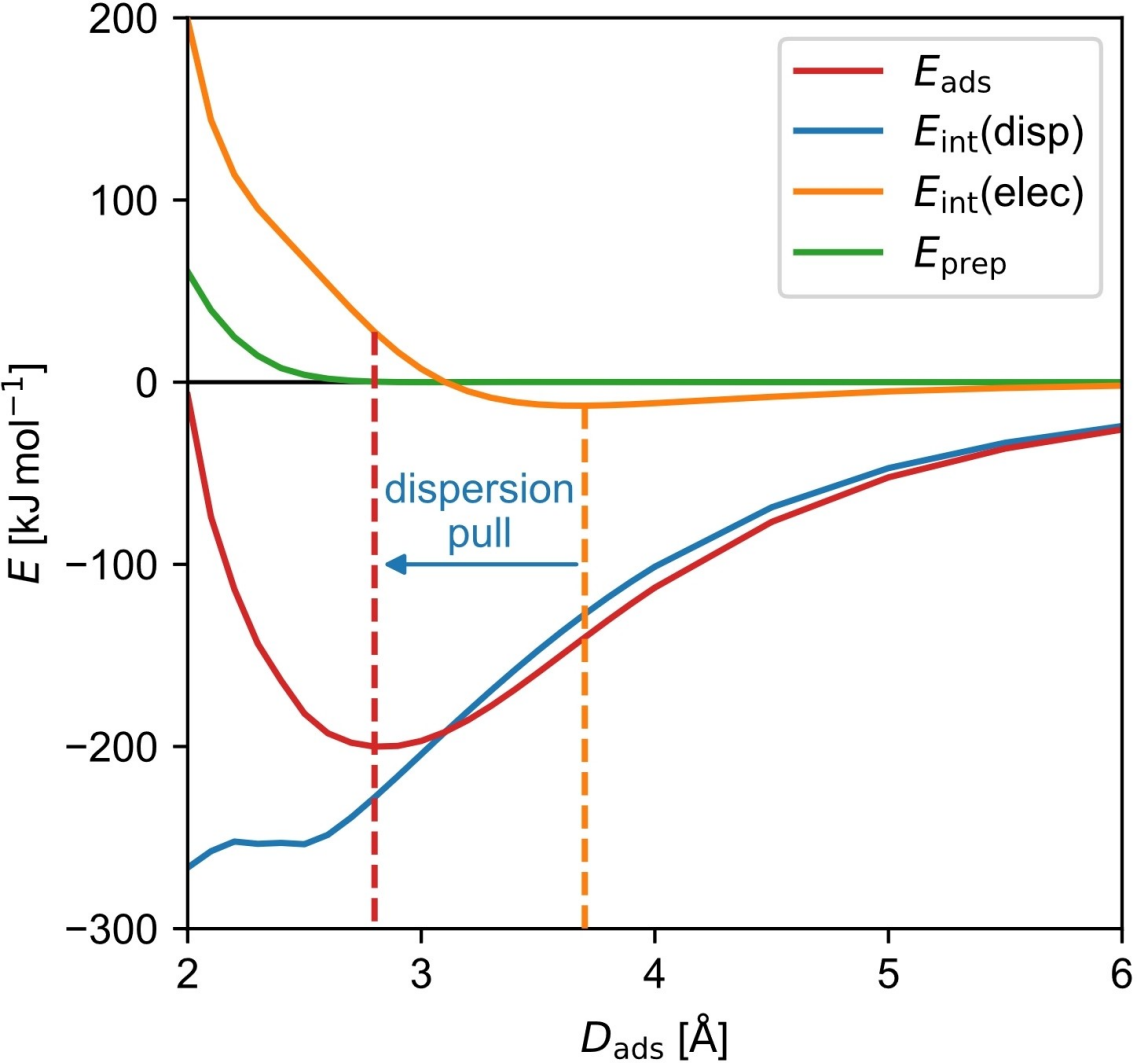
Curve of the adsorption energy *E*_ads_ and its decomposition into dispersion interaction energy *E*_int_(disp), electronic interaction energy *E*_int_(elec), and preparation energy *E*_prep_ in dependence on the adsorption height *D*_ads_ for physisorbed **pht** obtained by model calculations where only the H atoms were left to freely optimize.

For physisorbed molecules, the stabilizing *E*_int_ results from a very large contribution of *E*_int_(disp) (*E*_int_(disp)/*E*_int_=106 to 111 %; Supporting Information Table S2) which more than counterbalances a positive *E*_int_(elec) value (Figure 1, right). A destabilizing electronic interaction arises since the molecules are pulled towards the surface by the dispersion interaction between the molecule and the surface (dispersion pull, Figure 2). This lowers the adsorption height significantly, while it enforces the electron density of the surface and that of the molecule to overlap, resulting in an increase of Pauli repulsion.^[18]^ However, the repulsion of the electron density is compensated by the gain in dispersion energy up to the respective equilibrium.^[18]^ Note that the non‐smooth behavior of *E*_int_(disp) at lower adsorption heights stems from a change in the coordination numbers used in the evaluation of the DFT−D3 dispersion energy – an intrinsic parameter in this method.^[31]^

From a chemical point of view, this represents a unique bonding situation for physisorbed molecules, as the surface‐molecule bond is not electronically favored due to the dispersion pull. Still, there is a change in the electron densities because of the Pauli repulsion, also known as the Pauli pushback effect.[^13^, ^52^] It can therefore be assumed that this alters both the properties of the molecule and those of the surface.

**Figure 3:**
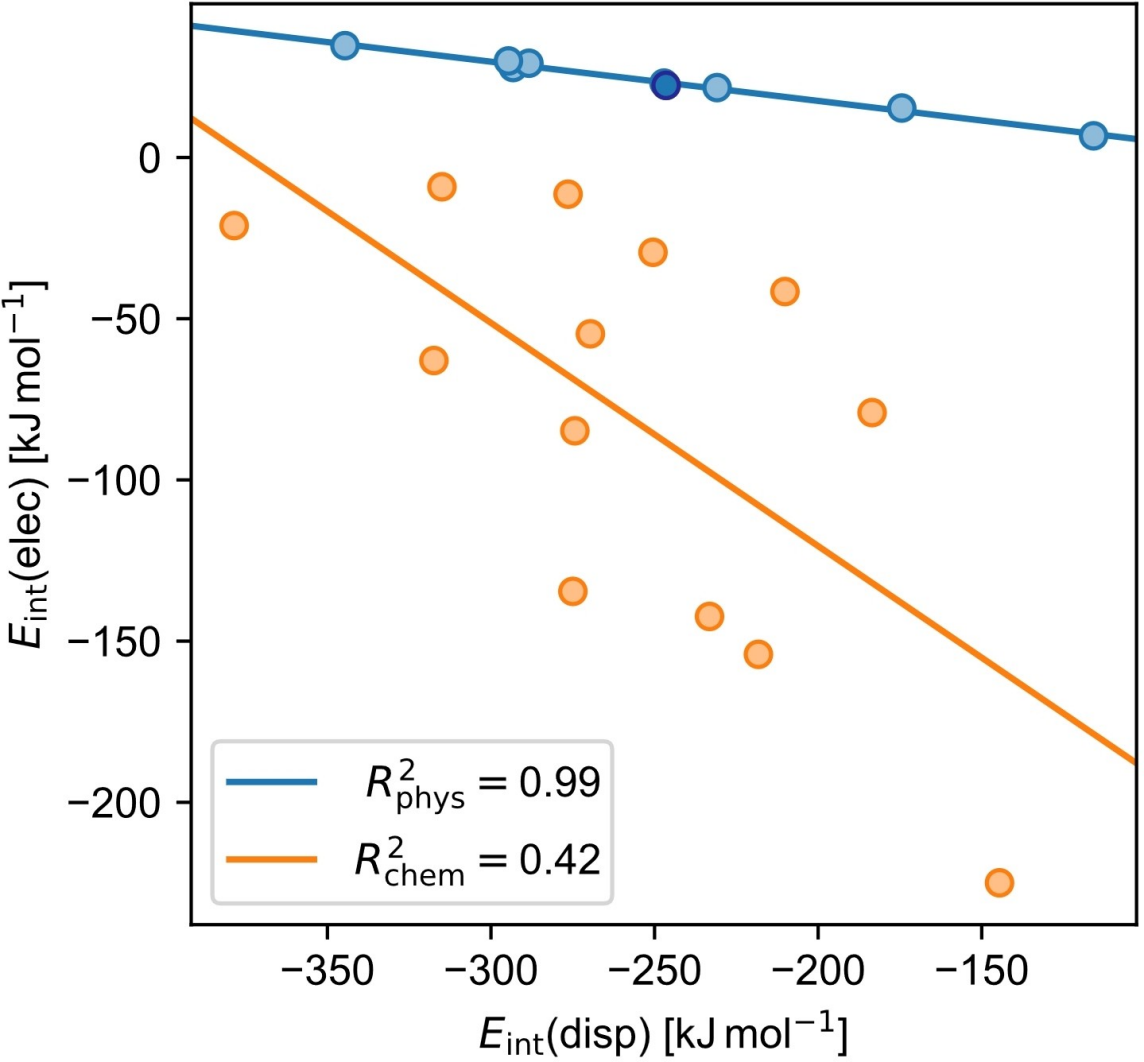
Correlation plot of the dispersion interaction energy *E*_int_(disp) and the electronic interaction energy *E*_int_(elec) for chemisorbed (chem, orange) and physisorbed molecules (phys, light blue). Note that **anc** is highlighted among the physisorbed molecules by a dark blue color.

Interestingly, the repulsive *E*_int_(elec) correlates with *E*_int_(disp) for physisorbed molecules (R^2^_phys_=0.99; Figure 3), which is not the case for chemisorbed molecules (R^2^_chem_=0.42; Figure 3). At least for physisorbed molecules, this means *E*_int_(disp) is a direct indicator of the repulsive energy due to Pauli repulsion, another hint that the mechanism of dispersion pull is decisive.

In general, it was found that *E*_int_(disp) correlates with the number of atoms in the molecule (R^2^_phys_=0.99 and R^2^_chem_=1.00; Supporting Information Figure S2) and thus the size of the molecule. This observed correlation is expected due to the atomic pairwise DFT−D3 dispersion correction scheme used.^[31]^ Chemisorbed molecules have a more negative *E*_int_(disp) than similarly sized physisorbed molecules, simply because they are closer to the surface as shown in section 4.2.1. This leads to a smaller distance in the pairwise dispersion method and therefore to a more stabilizing energy.

#### 4.1.3 Deformation of Adsorbed Molecules from Preparation Energy

Another energetic quantity derived from the decomposition of *E*_ads_ is the preparation energy (*E*_prep_), which indicates the extent of deformation of the molecule and surface and thus if a bond is formed (Scheme 2a).

**Figure 4:**
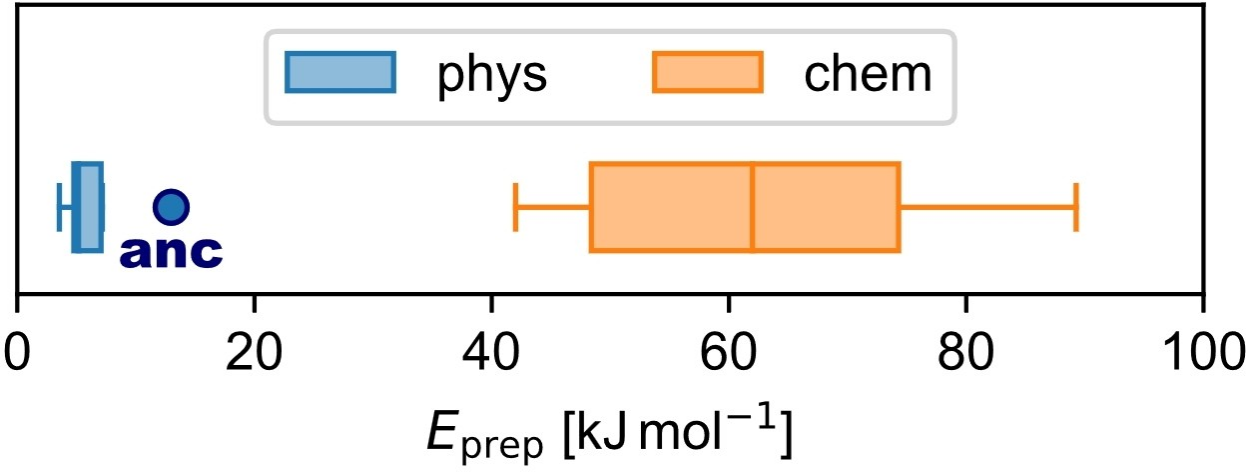
Box plot of the preparation energy *E*_prep_ for chemisorbed (chem, orange) and physisorbed molecules (phys, light blue). Note that **anc** is highlighted among the physisorbed molecules by a dark blue color.

All chemisorbed molecules have significant *E*_prep_ values (42 to 89 kJ/mol; Figure 4), which corresponds to the deformation of the molecule and the surface from their optimal gas phase geometry in the bond formation process. Both the preparation energy of the molecule *E*_prep_(mol) and the surface *E*_prep_(surf) play an important part in total (Supporting Information Figure S3). This shows that both the molecule and the surface are deformed as a chemical bond is formed between them. The proportion of *E*_prep_(mol)/*E*_prep_ is larger than 50 % (59 to 78 %, Supporting Information Table S2), showing a larger contribution of the molecular deformation. However, no correlation between *E*_prep_ or its decomposed subparts and other interface properties could be identified, as several factors influence the magnitude of *E*_prep_ like bending of the C‐H bonds or corrugation of the backbone, which in turn is dependent on several factors like the number of C‐H bonds or the degree of annulation.

In contrast, all physisorbed molecules show a much lower *E*_prep_ close to zero (4 to 7 kJ/mol, except for **anc** with 13 kJ/mol; Figure 4). Additionally, the proportion *E*_prep_(mol)/*E*_prep_ is smaller than 50 % (31 to 43 %, except for **anc** with 51 %, Supporting Information Table S2). This means that the physisorbed molecules lay flat on the surface with approximately their gas phase geometry and that no chemical bond to the surface is formed. In contrast, the surface atoms get distorted due to Pauli‐repulsion between the molecular electron density and the surface electron density. It should be noted that *E*_prep_ close to zero is only the case because the physisorbed molecules investigated here are already planar in the gas phase. In general, non‐planar molecules that physisorb can also be strongly deformed to maximize the dispersion interaction with the substrate.

However, although **anc** was classified as a physisorbed molecule, it has a significantly larger *E*_prep_ compared to all other physisorbed molecules (Figure 4). Furthermore, the proportion *E*_prep_(mol)/*E*_prep_ is with 51 % exactly between physisorbed (<50 %) and chemisorbed molecules (>50 %). This already shows that **anc** is an outlier among physisorbed molecules and is located at the border between physisorption and chemisorption, which will be also apparent later in other interface properties. Indications of the reason why **anc** is an outlier, will be discussed at the end in section 4.3.1.

### 4.2 (B) Geometric Aspect of the Metal‐Organic Interface

Apart from the energetic situation at the metal‐organic interface, the geometry of the adsorbed molecules is important, as the first layer forms the basis for possible thin‐film growth.^[53]^ In general, all molecules adopt a (quasi−)planar adsorption structure with the π‐electron system being parallel to the Cu(111) surface. This is even true for molecules that are non‐planar in the gas phase (**HPL** and **DPD**).

#### 4.2.1 Adsorption Height

**Figure 5:**
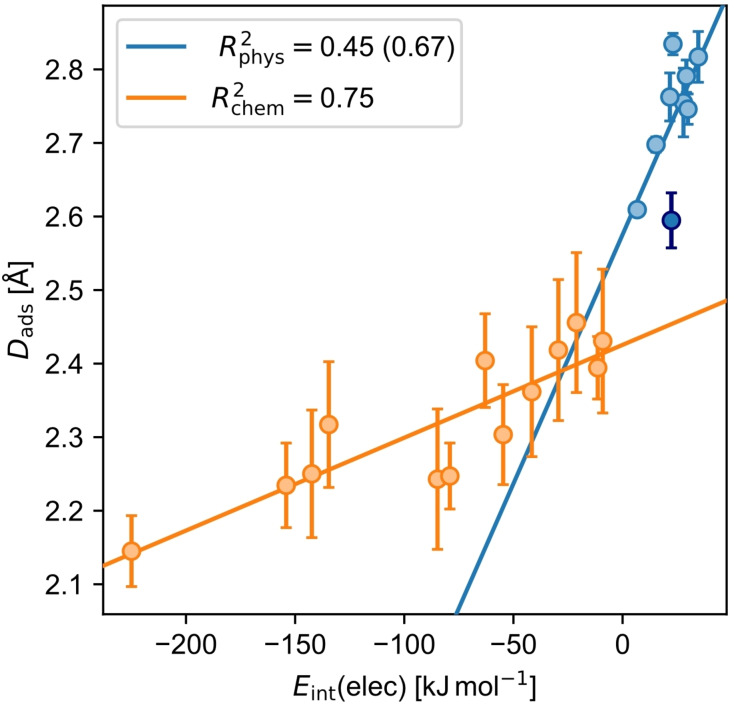
Correlation plot of the electronic interaction energy *E*_int_(elec) and the adsorption height *D*_ads_ with its mean absolute deviation *D*_ads_^MAD^ for chemisorbed (chem, orange) and physisorbed molecules (phys, light blue). Note that **anc** is highlighted among the physisorbed molecules by a dark blue color.

In this regard, an important parameter is the adsorption height (*D*_ads_). Here, the influence of the different adsorption regimes on the interface properties described above can be observed. Molecules that are chemisorbed are closer to the surface (2.15 to 2.46 Å; Figure 5), while physisorbed molecules are further away (2.59 to 2.83 Å; Figure 5).

In addition, chemisorbed molecules exhibit a large deviation in the adsorption height of the individual carbon atoms, from which *D*_ads_ is defined as the mean, as shown by large MAD bars of *D*_ads_ (*D*_ads_^MAD^, Figure 5). This indicates corrugation of the chemisorbed molecules, which is consistent with the large *E*_prep_ of chemisorbed molecules (Figure 4). However, *D*_ads_^MAD^ does not correlate to any other interface property since it is highly dependent on the structure, e. g., the degree of annulation. In contrast, this corrugation is not as visible for physisorbed molecules, which have much smaller MAD bars (Figure 5). This means they lay flat on the surface, which is consistent with the significantly smaller *E*_prep_ of physisorbed molecules.

Interestingly, a correlation between *D*_ads_ and *E*_int_(elec) could be observed (R^2^_phys_=0.45 and R^2^_chem_=0.75; Figure 5). For chemisorbed molecules, the larger the stabilizing *E*_int_(elec) is, the closer the molecule is to the surface, which seems chemically intuitive for a bond formation. Similarly, physisorbed molecules are further away from the surface the more destabilizing *E*_int_(elec) is. Since for physisorbed molecules, the repulsive *E*_int_(elec) is directly proportional to *E*_int_(disp) (Figure 3), this also means that physisorbed molecules are further away from the surface the larger *E*_int_(disp) and thus the larger the molecule is (Supporting Information Figure S2). Apparently, the size of the π‐electron system is a crucial factor for the adsorption height of physisorbed molecules. It seems that for the interaction between a π‐electron system and a surface, the Pauli repulsion is growing more quickly with size than dispersion attraction (Supporting Information Figure S4). In the extreme case of the infinitely large alternant π‐electron system graphene, *D*_ads_ reaches a maximum limit of 3.14 Å,[^20^, ^54^] which is notably larger than *D*_ads_ of the molecules investigated here. Surprisingly, this effect does have only a small influence on the correlation for the chemisorbed molecules. This is probably because smaller molecules generally have a lower aromaticity and therefore interact electronically stronger with the surface, which overlay the intrinsically smaller Pauli repulsion.

Like above, **anc** can be identified as an outlier here. It is the physisorbed molecule that is closest to the surface and in the same *D*_ads_ range as **bnz**, the smallest molecule investigated. Compared to physisorbed molecules of similar size, it is much closer to the surface. Removing it from the set of physisorbed molecules improves the correlation (R^2^_phys_ increases from 0.45 to 0.67).

#### 4.2.2 Bending of Adsorbed Molecules

**Figure 6:**
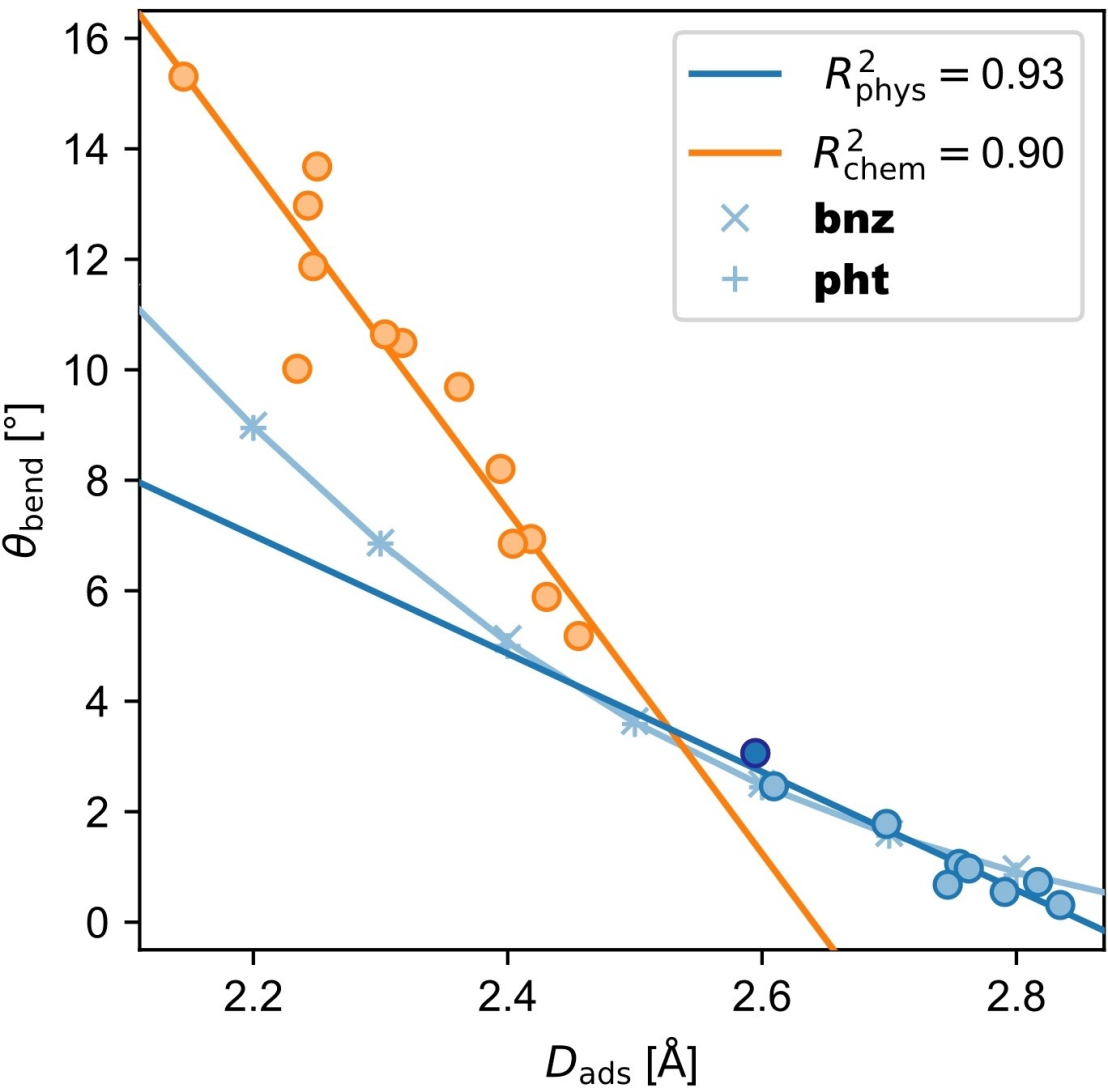
Correlation plot of the adsorption height *D*_ads_ and the C−H bending angle θ_bend_ for chemisorbed (chem, orange) and physisorbed molecules (phys, light blue). Note that **anc** is highlighted among the physisorbed molecules by a dark blue color.

Chemisorbed molecules distort upon adsorption. This bending of the molecules is not only partly reflected in the deviation of adsorption height but can also be confirmed by the bending angle of the C−H bonds (θ_bend_). Chemisorbed molecules show a larger θ_bend_ (5.2 to 15.3°; Figure 6) than physisorbed molecules (0.3 to 3.1°; Figure 6), again confirming the planarity of physisorbed and the bending of chemisorbed molecules due to formation of a chemical bond. Additionally, it is consistent with the larger *E*_prep_ of chemisorbed molecules. The magnitude of how much a molecule is bent correlates to *D*_ads_ of the molecule (R^2^_phys_=0.93 and R^2^_chem_=0.90; Figure 6). The closer the molecule to the surface, the larger θ_bend_.

There are two effects that contribute to bending of the C−H bonds: First, as there is a smaller dispersion attraction between the H atoms and the surface than between the C atoms and the surface, the C atoms will be pulled closer to the surface and the H atoms are (Pauli) repelled by the electron density of the surface. Second, partial hybridization between the sp^2^‐hybridized C atoms of the π‐electron system and the surface can occur, leading to a larger sp^3^‐like contribution, which results also in bending of the C−H bonds. This hybridization between the Cu surface atoms and the C atoms was also shown in earlier work by quantitative bonding analysis methods.[^18^, ^20^]

Since the physisorbed molecules do not form a chemical bond to the surface and thus do not hybridize with it, only the first effect is relevant. To estimate how important the second effect is for chemisorbed molecules, we did the following computational experiment: The C atoms of **bnz** and **pht**, two molecules of different size that physisorb, were fixed at different heights above a frozen Cu(111) surface (corresponding to *D*_ads_) and the H atoms were left to freely optimize. First of all, it shows that θ_bend_ is independent of the size of the molecule as the data points of **bnz** and **pht** strongly overlap (Figure 6). The curve seems to depend only on *D*_ads_ and not on the π‐electron system, which confirms the first effect. It should be noted that it is not a linear curve, but the physisorbed molecules are still in a rather linear regime at their equilibrium position, which justifies the linear fit.

Looking at the chemisorbed molecules, all of them show a larger θ_bend_ than the curve of **bnz** and **pht** (Figure 6). This shows that in the case of chemisorbed molecules, the second effect is more important for the magnitude of θ_bend_. This also explains the two different slopes found between physisorbed and chemisorbed molecules as there are two different mechanisms for bending of the C−H bonds.

### 4.3 (C) Electronic Aspect of the Metal‐Organic Interface

As mentioned in the introduction, the metal‐organic interface determines crucial performance parameters of electronic devices like the work function of the metal substrate (Φ) and charge carrier injection rates.[^12^, ^13^, ^14^] A key role is attributed to a vertical surface dipole. Such a dipole is already present on a clean metal surface, as electron density at the surface is spilling out into the vacuum.[^12^, ^13^, ^14^] In simplified terms, the adsorption of a molecule on this surface can now lead to two effects: On the one hand, if the molecule shows a strong electronic interaction with the surface (chemisorption), charge could be transferred from the surface to the molecule, increasing the vertical surface dipole. This would increase Φ and lead to an improved hole injection.[^12^, ^13^, ^14^] On the other hand, the surface dipole of the clean surface could be reduced due to the Pauli pushback effect: As the dispersion interaction pulls the molecule towards the surface, electron density between the surface and the molecule is pushed back due to Pauli repulsion. This decreased surface dipole would decrease Φ and lead to an improved electron injection.[^12^, ^13^, ^14^, ^52^] However, the exact behavior of the surface dipole during adsorption can be rather complex.[^12^, ^13^, ^14^]

#### 4.3.1 Charge Transfer

**Figure 7:**
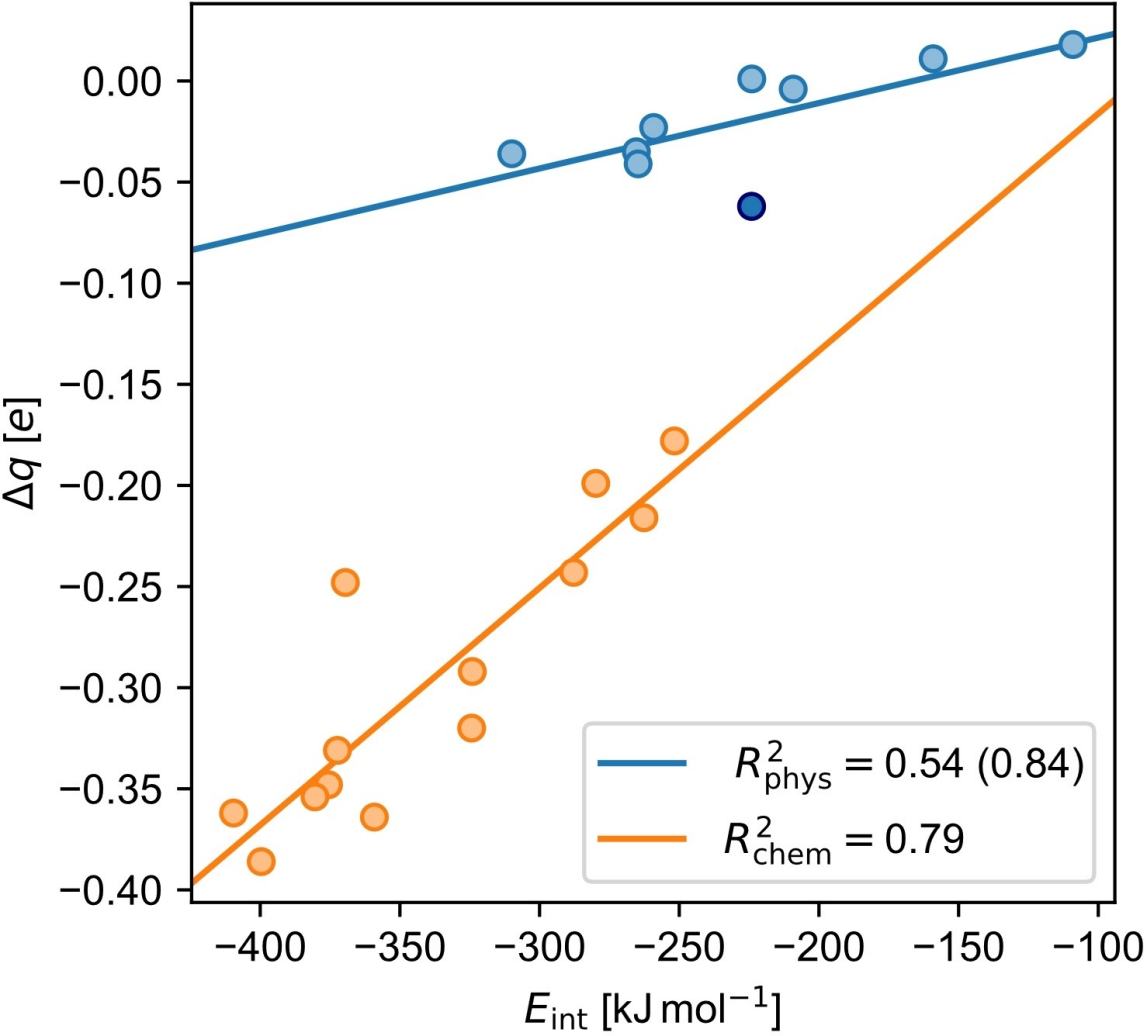
Correlation plot of the interaction energy *E*_int_ and charge transfer Δ*q* for chemisorbed (chem, orange) and physisorbed molecules (phys, light blue). Note that **anc** is highlighted among the physisorbed molecules by a dark blue color.

To disentangle the two effects on the vertical surface dipole, we will first look at the charge transfer (Δ*q*). This will also help to understand the direction of the formed bond, i. e. whether the lowest unoccupied molecular orbital (LUMO) or the highest occupied molecular orbital (HOMO) of the molecule participates in the bond. The difference between chemisorption and physisorption can be observed there again: Chemisorbed molecules have a significantly larger Δ*q*, receiving electrons from the surface (−0.18 to −0.39 e, Figure 7). This corresponds to an interaction between the surface and the LUMO, which was also observed in previous work.[^16^, ^17^, ^18^, ^20^, ^21^] In contrast, physisorbed molecules pick up nearly no charge upon adsorption (+0.02 to −0.06 e, Figure 7).

Here, a correlation between Δ*q* and the total *E*_int_ could be found (R^2^_phys_=0.54 and R^2^_chem_=0.79; Figure 7). For both adsorption behaviors, the stronger *E*_int_ with the surface is, the larger Δ*q* is. However, for chemisorbed molecules, the correlation has a larger slope, meaning that a change in *E*_int_ has a larger influence on Δ*q*. At a first glance it seems unreasonable why Δ*q* correlates to *E*_int_ instead of *E*_int_(elec), because the latter seems decisive for the bond formation and therefore Δ*q*. However, due to the aforementioned dispersion pull, the molecule gets closer to the surface and therefore forces electron density onto the molecule. Thus, the sum of *E*_int_(disp) and *E*_int_(elec), *E*_int_, is decisive. As a result, the increase in Δ*q* for physisorbed molecules can be explained, too, since *E*_int_ is just made up by *E*_int_(disp) and a larger dispersion pull enforces more charge transfer.

Once again, **anc** can be found as an outlier among physisorbed molecules. It receives the most charge from the surface among physisorbed molecules and more than physisorbed molecules of comparable size and *E*_int_. If it is removed from the set of physisorbed molecules, R^2^_phys_ increases from 0.54 to 0.84, improving the correlation.

Finally, the probable reason why **anc** is an outlier among physisorbed molecules can be addressed. The most intuitive approach is to compare it to its physisorbed isomer **pht**. The decomposed adsorption curves reveal that the repulsive *E*_int_(elec) of **anc** has a slightly smaller slope, resulting in different minima of *E*_ads_ (Supporting Information Figure S5). Especially the corrugation of the molecule and the surface, which is not accounted for in the adsorption curves, seems to be decisive as it further increases this effect. Although the differences in energy are rather minor, given the flat curve of the adsorption energy, it seems to be sufficient so that **anc** remains closer to the surface. Furthermore, **anc** shows a larger charge transfer than expected, which is additionally indicated by the LUMO being located near the Fermi energy (Supporting Information Figure S6). Those are important findings, as **anc** lies exactly at the boundary between what we have defined as physisorption and chemisorption regimes and could be used as a reference point.

#### 4.3.2 Work Function Change

**Figure 8:**
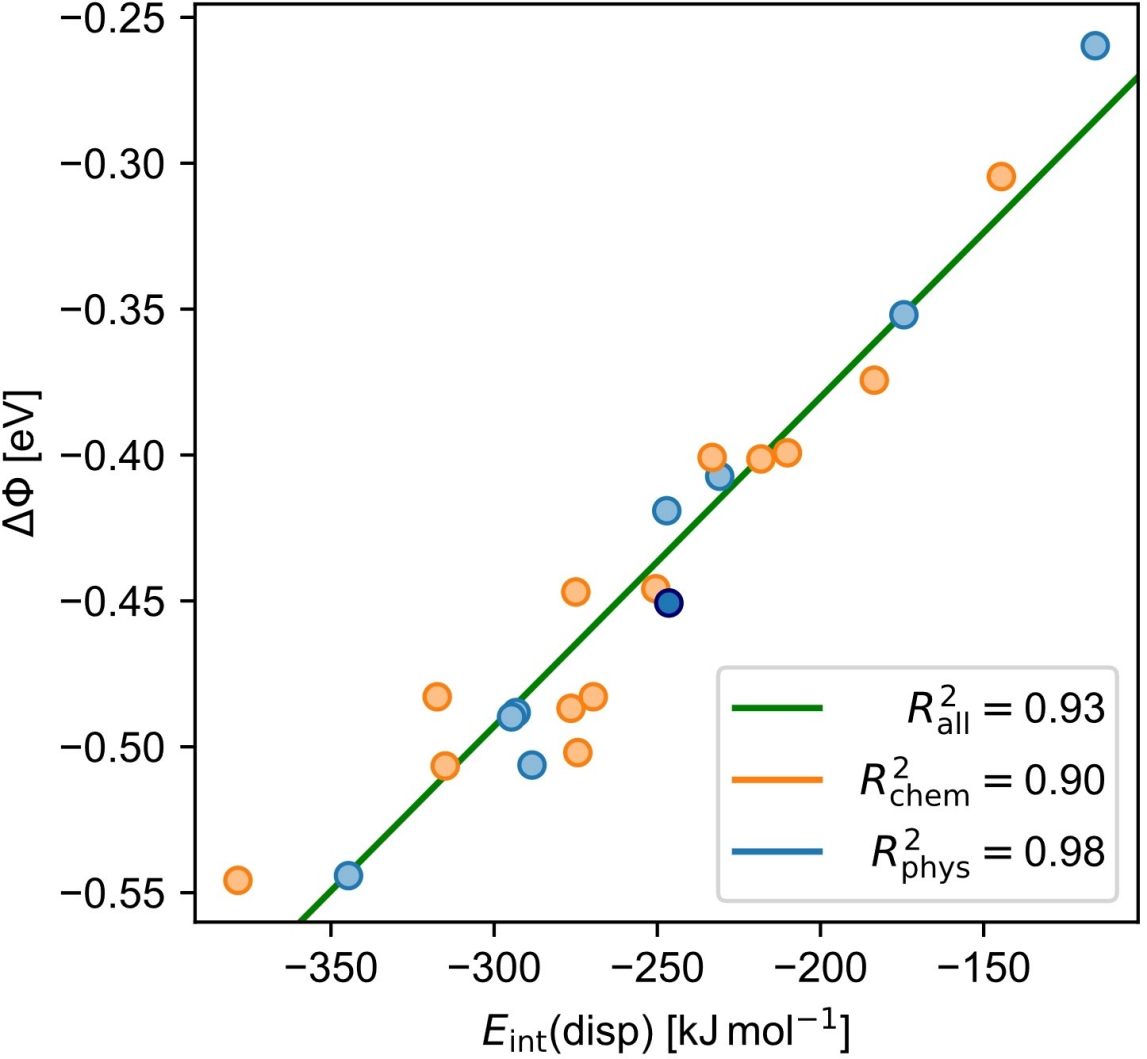
Correlation plot of the dispersion interaction energy *E*_int_(disp) and change in work function ΔΦ for chemisorbed (chem, orange) and physisorbed molecules (phys, light blue). The linear fit for all molecules is shown in green. Note that **anc** is highlighted among the physisorbed molecules by a dark blue color.

Which of the two effects mentioned above is dominant can be determined directly from the change in working function of the Cu(111) surface with an adsorbed molecule relative to the clean surface (ΔΦ). It is noticeable that the change is negative (−0.26 to −0.55 eV; Figure 8) regardless of whether the molecule is chemisorbed or physisorbed. Based on the reduced Φ, it can be concluded that the Pauli pushback effect is dominant in both cases and overcompensates a charge transfer.[^12^, ^13^, ^14^, ^52^] This has already been noted in previous work.[^16^, ^17^, ^20^]

Interestingly, a correlation between ΔΦ and *E*_int_(disp) can be observed (R^2^_all_=0.93; Figure 8), again independent of the two adsorption regimes. In both cases, ΔΦ is larger if *E*_int_(disp) is larger. This can be explained by the observation made for physisorbed molecules (Figure 3): *E*_int_(disp) is a direct indicator of Pauli repulsion, which dominates the change in the surface dipole by the pushback effect. Apparently, this is transferable to chemisorbed molecules, where the dispersion pull on the molecule enforces overlap of electron density beyond the equilibrium structure resulting from purely electronic interactions. Therefore, this dispersion‐forced Pauli repulsion is the actual quantity that correlates with the change in surface dipole and ΔΦ.

Although the overall correlation between ΔΦ and *E*_int_(disp) is independent of the adsorption behavior, there is still a difference between physisorbed and chemisorbed molecules of same size if Figure 5 and Supporting Information Figure S2 are brought back to mind. Chemisorbed molecules are generally closer to the surface than physisorbed molecules and have therefore also a larger *E*_int_(disp). Thus, molecules of same size, like isomer pairs with non‐alternant and alternant topologies, still show a different ΔΦ, as was also observed experimentally for azulene and naphthalene (ΔΦ_exp_=−1.07 vs. −0.75 eV)[^16^, ^17^] or azupyrene and pyrene (ΔΦ_exp_=−1.18 vs. −0.86 eV)^[20]^.

It should be noted that the influence of the charge transfer due to bond formation may be visible in the coefficient of determination of chemisorbed molecules. It is worse (R^2^_chem_=0.90; Figure 8) than that for physisorbed molecules (R^2^_phys_=0.98; Figure 8). A major part of this should be due to charge transfer, as it is changes, even only slightly, the vertical dipole moment and consequently ΔΦ. Therefore, it leads to a broader scattering of ΔΦ for chemisorbed molecules, regardless of the size of the molecule and thus *E*_int_(disp), reducing the correlation with *E*_int_(disp).

## 5 Conclusions

In conclusion, we investigated Cu(111)‐organic interfaces using *in‐silico* screening of a set of 11 non‐alternant and 11 alternant π‐electron systems using first principle DFT calculations. This enabled us to study the two different adsorption regimes, physisorption and chemisorption, as well as different interaction strengths within one regime. In this way, we understand what the differences in interface properties between the two adsorption regimes are and how the different interface properties are connected to each other.

A generalized summary of the differences in properties between Cu(111)‐organic interfaces with chemisorbed and with physisorbed molecules is shown in Table 2. We found that all non‐alternant molecules are chemisorbed on the Cu(111) surface, which might be an important finding for interface design. Additionally, the two longer alternant acenes **ttc** and **pnc** were chemisorbed. Chemisorption is characterized by: (1) stabilizing electronic interactions with surface‐to‐molecule charge transfer (negative *E*_int_(elec), large Δ*q*); (2) considerable deformation of surface and molecule (large *E*_prep_, large *D*_ads_^MAD^, large θ_bend_); and (3) low adsorption height (small *D*_ads_).

**Table 2:** Generalized summary of the differences between the investigated Cu(111)‐organic interface properties of chemisorbed and of physisorbed molecules.

Properties	Chemisorption	Physisorption
*E*_ads_	no difference^[a]^	
*E*_int_(elec)	<0	>0
*E*_int_(disp)/*E*_int_	<100 %	>100 %
*E*_prep_	>0	≈0^[b]^
*E*_prep_(mol)/*E*_prep_	>50 %	<50 %^[b]^
*D*_ads_	<2.5 Å	>2.5 Å
*D*_ads_^MAD^	>0	≈0
θ_bend_	>0	≈0
Δ*q*	<0	≈0
ΔΦ	no difference^[a]^	

^[a]^ Molecules of similar size that differ in the adsorption regime might show a more negative *E*_ads_ and a more negative ΔΦ. ^[b]^ This is only true for molecules that are already planar in the gas phase. In general, non‐planar molecules that physisorb can be strongly deformed to maximize the dispersion interaction with the substrate.

The interface properties of physisorbed molecules are dominated by the mechanism of dispersion pull: Due to dispersion interactions, the molecules are pulled towards the surface, forcing the electron density of the molecule and the surface to overlap. This leads to a significant amount of Pauli repulsion between the molecule and the surface, which is a unique bonding situation as it is an electronically repulsive bond. This is accompanied by an increase in adsorption height for larger physisorbed molecules. Although this seems counterintuitive as the dispersion attraction increases with size of the π‐electron systems, the Pauli repulsion enhances even more, explaining this finding.

Although the dispersion pull was mainly observed for physisorbed molecules, it also plays a decisive role for chemisorbed molecules as was shown in the change of work function ΔΦ. Both physisorbed and chemisorbed molecules showed a reduction in the work function, which means that the vertical surface dipole moment is getting smaller, corresponding to domination of the Pauli pushback effect over charge transfer effects.[^12^, ^13^, ^14^, ^52^]

Furthermore, we found that there are chemically intuitive and quantitative correlations between the interface properties. Thus, they also come as a “fixed set” of properties for an individual molecule, e. g., a molecule that shows strong stabilizing electronic interactions with the surface is also closer to the surface, has a larger bending of the C−H bonds, and receives more charge from the surface.

This provides important insights into how the adsorption regime decisively influences the interface properties. Together with the physicochemical relations found within a regime and the derived concepts, this will contribute to the understanding and improvement of metal‐organic interfaces in organic electronic devices.

## Conflict of Interests

The authors declare no conflict of interest.

## Supporting information

As a service to our authors and readers, this journal provides supporting information supplied by the authors. Such materials are peer reviewed and may be re‐organized for online delivery, but are not copy‐edited or typeset. Technical support issues arising from supporting information (other than missing files) should be addressed to the authors.

Supporting Information

## Data Availability

The raw data underlying this study are available on Zenodo (DOI: https://doi.org/10.5281/zenodo.14416729).
