# Supplementary material for: Unveiling Correlations in Metal‐Organic Interface Properties: A Computational Exploration of Alternant and Non‐Alternant π‐Electron Systems
Source: Chempluschem. 2025 Mar 20;90(6):e202400771. doi: 10.1002/cplu.202400771 (PMC12143461; doi:10.1002/cplu.202400771)
Supplement: Supplementary file 1 — Supporting Information [file CPLU-90-e202400771-s001.pdf]

ChemPlusChem

Supporting Information

Unveiling Correlations in Metal-Organic Interface Properties: A Computational Exploration of Alternant and Non-Alternant π-Electron Systems

Jakob Schramm and Ralf Tonner-Zech*

1 Detailed Theoretical Background

1.1 (A) Energetic Aspect

The **adsorption energy** (E_{ads}) is calculated in a supermolecular approach as the difference between the energy of the optimized adsorbate-surface $\left(E_{\text{asc}}^{\text{opt}}\right)$ complex and the energies of the independently optimized molecule $\left(E_{\text{mol}}^{\text{opt}}\right)$ and surface $\left(E_{\text{surf}}^{\text{opt}}\right)$.

$$E_{\text{ads}} = E_{\text{asc}}^{\text{opt}} - \left(E_{\text{mol}}^{\text{opt}} + E_{\text{surf}}^{\text{opt}}\right)$$

Alternatively, E_{ads} is decomposed into a **preparation energy** $\left(E_{\text{prep}}\right)$ and an **interaction energy** (E_{int}).

$$E_{\text{ads}} = E_{\text{prep}} + E_{\text{int}}$$

E_{prep} is calculated as the respective difference between the energies of the independent fragments frozen in the adsorbate-surface complex geometry (adsorbate molecule $\left[E_{\text{mol}}^{\text{frz}}\right]$ and surface $\left[E_{\text{surf}}^{\text{frz}}\right]$) and the energies of the independently optimized fragments (adsorbate molecule $\left[E_{\text{mol}}^{\text{opt}}\right]$ and surface $\left[E_{\text{surf}}^{\text{opt}}\right]$).

$$E_{\text{prep}} = \left(E_{\text{mol}}^{\text{frz}} - E_{\text{mol}}^{\text{opt}}\right) + \left(E_{\text{surf}}^{\text{frz}} - E_{\text{surf}}^{\text{opt}}\right)$$

Thus, E_{prep} is further decomposed into a **molecular preparation energy** $\left[E_{\text{prep}}(\text{mol})\right]$ and a **surface preparation energy** $\left[E_{\text{prep}}(\text{surf})\right]$.

$$E_{\text{prep}} = E_{\text{prep}}(\text{mol}) + E_{\text{prep}}(\text{surf})$$

E_{int} is calculated again in a supermolecular approach as the difference between the energy of the optimized adsorbate-surface $\left(E_{\text{asc}}^{\text{opt}}\right)$ complex and the energies of the independent fragments frozen in the adsorbate-surface complex geometry (adsorbate molecule $\left[E_{\text{mol}}^{\text{frz}}\right]$ and surface $\left[E_{\text{surf}}^{\text{frz}}\right]$).

$$E_{\text{int}} = E_{\text{asc}}^{\text{opt}} - \left(E_{\text{mol}}^{\text{frz}} + E_{\text{surf}}^{\text{frz}}\right)$$

As the additive DFT-D3(BJ) dispersion correction was used, E_{int} can further be decomposed into a **dispersion interaction energy** $[E_{\text{int}}(\text{disp})]$ and an **electronic interaction energy** $[E_{\text{int}}(\text{elec})]$.

$$E_{\text{int}} = E_{\text{int}}(\text{disp}) + E_{\text{int}}(\text{elec})$$

$E_{\text{int}}(\text{disp})$ then corresponds to the difference between the DFT-D3(BJ) dispersion energy of the optimized adsorbate-surface $\left(DE_{\text{asc}}^{\text{opt}}\right)$ complex and the DFT-D3(BJ) dispersion energies of the

independent fragments frozen in the adsorbate-surface complex geometry (adsorbate molecule $\left[DE_{\text{mol}}^{\text{frz}}\right]$ and surface $\left[DE_{\text{surf}}^{\text{frz}}\right]$).

$$E_{\text{int}}(\text{disp}) = DE_{\text{asc}}^{\text{opt}} - \left(DE_{\text{mol}}^{\text{frz}} + DE_{\text{surf}}^{\text{frz}}\right)$$

Similarly, $E_{\text{int}}(\text{elec})$ corresponds to the difference between the Kohn-Sham energy of the optimized adsorbate-surface $\left(KS_{\text{asc}}^{\text{opt}}\right)$ complex and the Kohn-Sham energies of the independent fragments frozen in the adsorbate-surface complex geometry (adsorbate molecule $\left[KS_{\text{mol}}^{\text{frz}}\right]$ and surface $\left[KS_{\text{surf}}^{\text{frz}}\right]$).

$$E_{\text{int}}(\text{elec}) = KS_{\text{asc}}^{\text{opt}} - \left(KS_{\text{mol}}^{\text{frz}} + KS_{\text{surf}}^{\text{frz}}\right)$$

Ultimately, E_{ads} is decomposed into the different contributions.

$$E_{\text{ads}} = E_{\text{prep}} + E_{\text{int}} = E_{\text{prep}}(\text{mol}) + E_{\text{prep}}(\text{surf}) + E_{\text{int}}(\text{disp}) + E_{\text{int}}(\text{elec})$$

This means that

- E_{ads} corresponds to the total energy gained by bonding between the relaxed molecule and the relaxed surface.
    - E_{prep} corresponds to the energy needed for the deformation of the system.
        - $E_{\text{prep}}(\text{mol})$ corresponds to the energy needed for the deformation of the adsorbed molecule.
        - $E_{\text{prep}}(\text{surf})$ corresponds to the energy needed for the deformation of the surface during adsorption.
    - E_{int} corresponds to the energy gained by interactions between the deformed molecule and the deformed surface
        - $E_{\text{int}}(\text{disp})$ corresponds to the energy gained by dispersion interactions between the deformed molecule and the deformed surface
        - $E_{\text{int}}(\text{elec})$ corresponds to the energy gained by electronic interactions between the deformed molecule and the deformed surface

1.2 (B) Geometric Aspect

The **adsorption height** (D_{ads}) is calculated as the difference between the mean of the z-coordinate of the C atoms of the molecule (z_i) and the z-coordinate of the uppermost layer of the independently optimized surface $\left(z_{\mathrm{surf}}^{\mathrm{opt}}\right)$. Note that the z-axis is perpendicular to the surface.

$$D_{\mathrm{ads}} = \frac{1}{N_{\mathrm{C}}} \sum_{i}^{N_{\mathrm{C}}} z_i - z_{\mathrm{surf}}^{\mathrm{opt}}$$

The **mean absolute deviation of the adsorption height** $\left(D_{\mathrm{ads}}^{\mathrm{MAD}}\right)$ is calculated using the standard definition.

$$D_{\mathrm{ads}}^{\mathrm{MAD}} = \frac{1}{N_{\mathrm{C}}} \sum_{i}^{N_{\mathrm{C}}} \left| z_i - z_{\mathrm{surf}}^{\mathrm{opt}} - D_{\mathrm{ads}} \right|$$

The **bending angle** (θ_{bend}) is calculated as the mean of the individual C-H bending angles, which are calculated as the arcsine of the fraction of the displacement along the z-axis over the C-H bond length (d_{CH}).

$$\theta_{\mathrm{bend}} = \frac{1}{N_{\mathrm{CH}}} \sum_{i}^{N_{\mathrm{CH}}} \arcsin\left(\frac{z_{C_i} - z_{H_i}}{d_{C_i H_i}}\right)$$

1.3 (C) Electronic Aspect

The **charge transfer** (Δq) is calculated by summation of all partial (Hirshfeld) charges of the atoms of the molecule (q_i), as this is the total difference to the gas phase, where it is neutral.

$$\Delta q = \sum_{i}^{N_{\mathrm{mol}}} q_i$$

The **change of work function** $(\Delta \Phi)$ is calculated as the difference between the work function of the adsorbate-surface complex (Φ_{asc}) and the work function of the pristine Cu(111) surface (Φ_{Cu}).

$$\Delta \Phi = \Phi_{asc} - \Phi_{Cu}$$

The work function (Φ) of any system is calculated as the difference between the electrostatic potential in the vacuum (V_{vac}) and the Fermi energy (E_{F}).

$$\Phi = V_{\mathrm{vac}} - E_{\mathrm{F}}$$

V_{vac} was determined from the averaged Hartree potential (in xy-plane) in the vacuum region, where the change of the slope was constant.

2 Correlation Table of Interface Properties

To identify relationships between different interface properties with each other, we looked at the R^2 of linear correlations (Figure S1). Since we expected different interface properties between chemisorbed and physisorbed molecules, we looked at them independently.

Correlations discussed in the main text are shown violet in Figure S1. Other correlations shown in Figure S1 can be explained by previously discussed observations:

- For both groups: As E_{prep} can be seen as a small distortion to E_{int}, the latter correlates with the sum of both (E_{ads}). Since E_{prep} is larger and shows a broader distribution for chemisorbed molecules, the correlation is worse. Furthermore, if one of them correlates with another interface property, the other will also (e.g. with Δq).
- For physisorbed molecules: As E_{int}(elec) correlates to E_{int}(disp), both also correlate to the sum of them (E_{int}). Thus, both also correlate to E_{ads}. Furthermore, if one of the energetic interface properties correlate to another geometric or electronic interface property, all will show correlations in the same magnitude (e.g. D_{ads}, Δq, or $\Delta\Phi$). Therefore, mainly the chemisorbed molecules were used to identify relations.

	E_{ads}	E_{prep}	E_{int}(disp)	E_{int}(elec)	E_{int}	D_{ads}	D_{ads}^{MAD}	θ_{bend}	Δq	$\Delta\Phi$
E_{ads}	100	3	100	99	100	46	47	39	49	97
E_{prep}	13	100	4	3	4	33	28	37	66	8
E_{int}(disp)	28	14	100	99	100	42	49	35	53	98
E_{int}(elec)	8	68	42	100	99	45	43	40	49	98
E_{int}	93	35	13	23	100	42	49	35	54	98
D_{ads}	1	65	62	75	3	100	4	93	0	35
D_{ads}^{MAD}	6	2	25	11	3	19	100	1	56	50
θ_{bend}	2	59	59	63	1	90	11	100	0	29
Δq	74	25	35	3	79	0	7	0	100	60
$\Delta\Phi$	12	19	90	56	3	56	23	51	24	100

Figure S1. Correlation table of interface properties with each other showing 100·R^2 for physisorbed (blue) and chemisorbed molecules (orange) independently. The color of a field corresponds to 100·R^2 (>90 full, <40 white, in between gradient). Correlations discussed in the main text are shown in violet.

3 Correlation between Number of Atoms and Dispersion Interaction

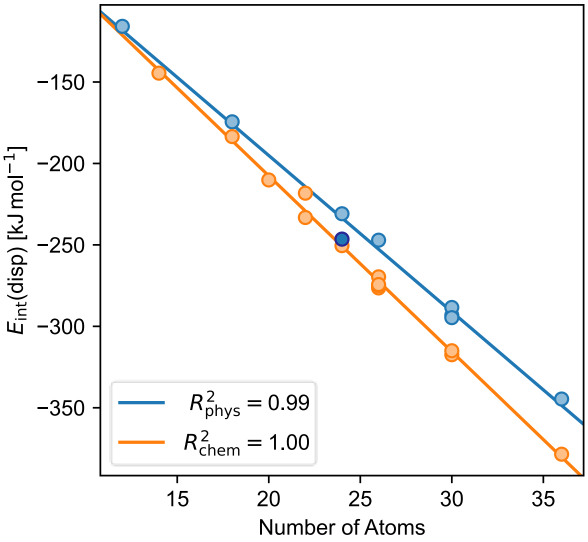

Figure S2. Correlation plot of the number of atoms and dispersion interaction energy E_{int}(disp) for chemisorbed (chem, orange) and physisorbed molecules (phys, light blue). Note that **anc** is highlighted among the physisorbed molecules by a dark blue color.

4 Decomposed Preparation Energy

Chemisorbed molecules have a significant larger E_{prep} than physisorbed ones, which are close to zero (see Figures 1 and 2 in the main text).

Considering the decomposition of E_{prep}, both the molecule and the surface are deformed for chemisorbed molecules (Figure S3). The proportion of the molecular deformation is larger (they lay on the right side of the black line in Figure S3).

For physisorbed molecules, there is also a contribution of both the molecule and the surface. However, the molecular deformation is smaller (they lay on the left side of the black line in Figure S3).

anc can be identified as an outlier among the physisorbed molecules since it is further away from them, and it lays on the black line in Figure S3.

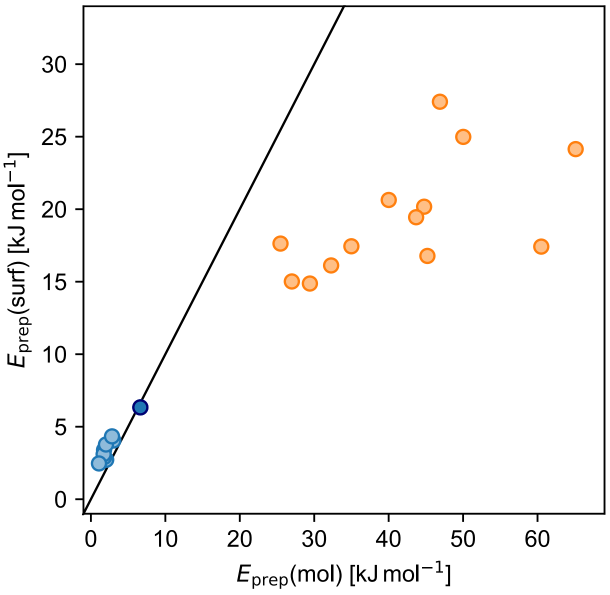

Figure S3. Correlation plot of the preparation energy of the molecule E_{prep}(mol) and the surface E_{prep}(surf) for chemisorbed (orange) and physisorbed molecules (light blue). The black line shows the 50% proportion of E_{prep}. Note that **anc** is highlighted among the physisorbed molecules by a dark blue color.

5 Size Dependent Repulsion of π-Electron Systems

It was found that D_{ads} of physisorbed molecules correlates with the repulsive E_{int}(elec) (Figure 5). Thus, D_{ads} also correlates with E_{int}(disp) and therefore with the size of the molecule. This means that larger molecules that physisorb are further away from the surface. To put this in perspective: the largest molecule that physisorbs, the infinitely large graphene, has a significantly larger D_{ads} (3.14 Å) compared to the molecules investigated here. Apparently, larger molecules seem to have intrinsically a larger growing Pauli repulsion than dispersion attraction.

To understand this in more detail, we did the following computer experiment: **bnz**, **pht**, and **pic** (3 physisorbed phenacenes with increasing size: 1, 3, and 5 benzene rings) were put in their relaxed gas phase structure above the relaxed Cu(111) surface at different distances (corresponding to D_{ads}) and single point calculations were done. In this way, no geometrical relaxation was allowed, canceling effects in the energy due to deformation (corresponding to E_{prep}=0). The resulting relative adsorption curves confirm that with increasing molecular size they have a larger D_{ads}. (Figure S4a). Note that as the molecule and surface are not allowed to relax, the minimum for each molecule shows a larger D_{ads} than found by a geometry optimization.

Additionally, one can look at the two contributions of E_{ads}, the attractive E_{int}(disp) and the repulsive E_{int}(elec) (Figure S4b). It is noticeable that the larger the molecule, the more destabilizing the Pauli repulsion [E_{int}(elec)], but also the more stabilizing the dispersion attraction [E_{int}(disp)]. As those effects might cancel each other, no conclusions can be made yet.

Thus, the fraction $|E_{int}$(elec)$|$ / $|E_{int}$(disp)$|$ was considered (Figure S4c). There, it can be seen that for an arbitrary D_{ads}, a larger molecule always has a higher proportion $|E_{int}$(elec)$|$ / $|E_{int}$(disp)$|$. Therefore, the point where the ratio is 1 and thus the minimum in E_{ads} is always at a larger D_{ads}.

This confirms that larger molecules have intrinsically a larger Pauli repulsion relative to its dispersion attraction, resulting in a larger D_{ads}.

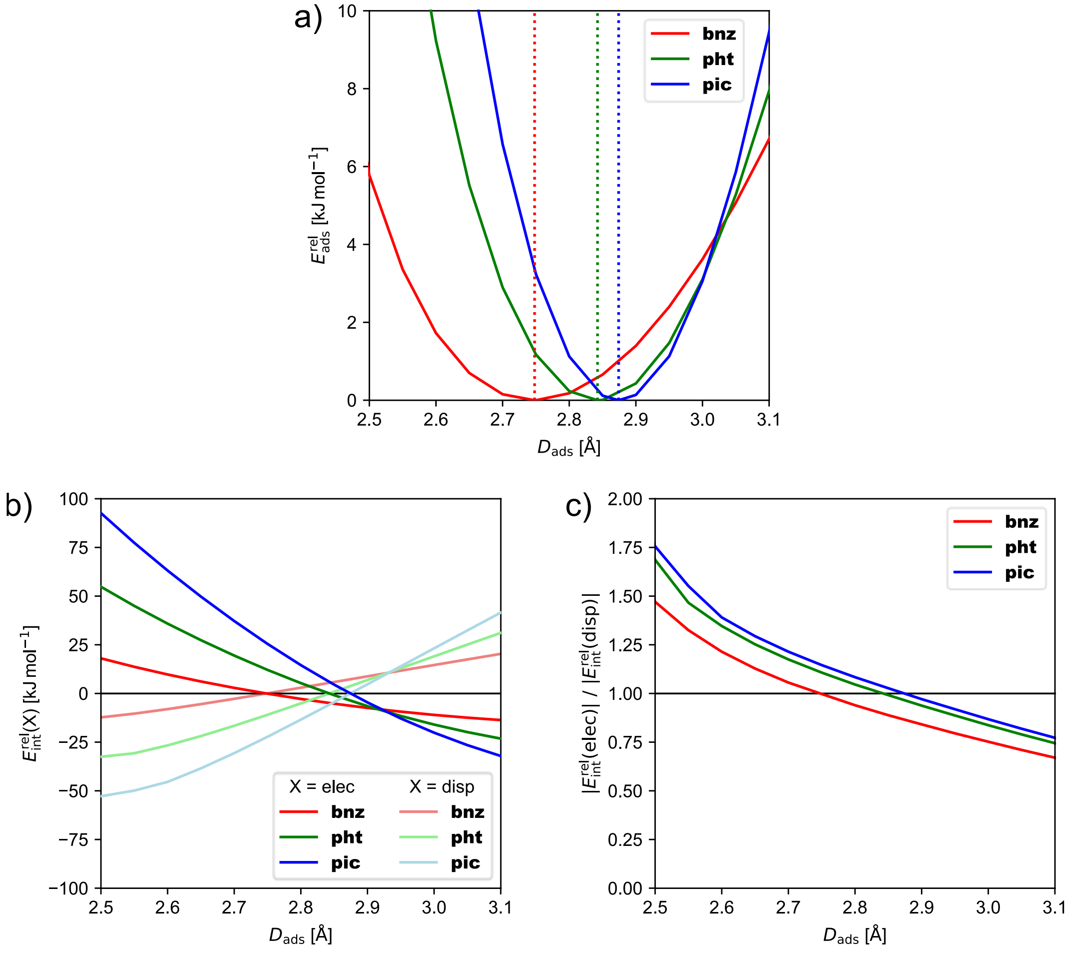

Figure S4. Computer experiment for the intrinsic Pauli repulsion for the increasing phenacene series **bnz**, **pht**, and **pic**. (a) Relative adsorption energy $E_{ads}{}^{rel}$ in dependence of the adsorption height D_{ads} with minima indicated by vertical lines. (b) Relative electronic and dispersion interaction energies $E_{int}{}^{rel}$(X) (with X = elec, or disp) in dependence of D_{ads}. (c) Absolute fraction of the relative electronic and relative dispersion interaction energy $|E_{int}{}^{rel}$(elec)| / $|E_{int}{}^{rel}$(disp)| in dependence of D_{ads}.

6 Comparison of the decomposed Adsorption Curves of anc and pht

To rationalize why **anc** is an outlier among the physisorbed molecules, we conducted a computer experiment in comparison to its isomer **pht**. We put both relaxed molecules at their favored adsorption site at various adsorption heights above a relaxed Cu(111) surface and kept the C and Cu atoms frozen so that only the H atoms could freely relax (the same as for section 4.2.2). From that it was possible to calculate the adsorption energy as well as its decomposition.

Comparing both molecules, all energy curves look very similar (Figure S5). For E_{prep} and E_{int}(disp), there is basically no difference. The only difference appears in E_{int}(elec) that also translates into E_{ads}. With decreasing adsorption height, **anc** is repulsed less than **pht**. Thus, it is not the minimum of the E_{int}(elec) curve, that differs (below -1 kJ/mol), but the slope of the repulsion that is smaller for **anc**, resulting at the minimum of E_{ads} in a difference of -4 kJ/mol (E_{ads} of **anc** is more negative and it is therefore stronger bonded than **pht**).

Still, this is not the full picture: Using a full geometry optimization, the difference in E_{ads} between **anc** and **pht** is -7 kJ/mol (Table S2). What was missing in the computer experiment is a relaxation of the molecule and the surface. Because of that, **anc** is deformed more than **pht** (difference in D_{ads}^{MAD} of 0.005 Å and difference in MAD of CC bond lengths of 0.004 Å). Although this leads to a larger E_{prep} (difference of +8 kJ/mol), it also enables **anc** to come closer to the surface, increasing E_{int}(disp) (difference of -16 kJ/mol). And here, the previously observed effect comes into play. Although **anc** is closer to the surface, the repulsive E_{int}(elec) is nearly the same as for **pht** (difference of +1 kJ/mol) because of the smaller slope for **anc**.

Although the discussed differences in energy between **anc** and **pht** seem all very small, they have a significant influence as **anc** is 0.17 Å closer to the surface than **pht** (Table S3). This, of course, also alters the electronic densities at the interface as discussed in section 4.3 of the main text.

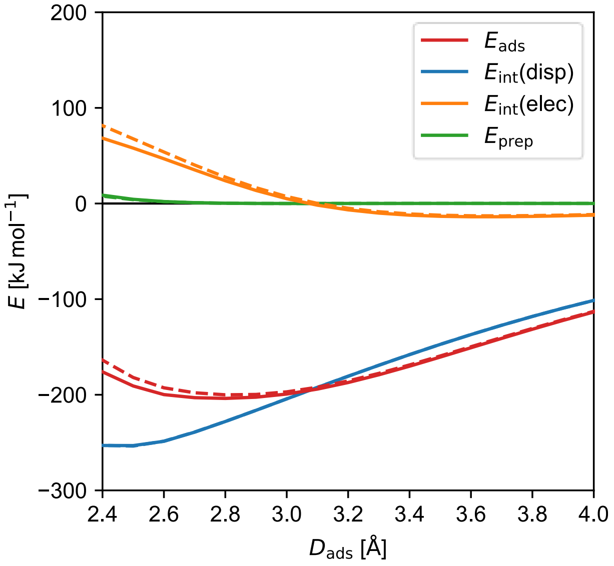

Figure S5. Curve of the adsorption energy E_{ads} and its decomposition into dispersion interaction energy E_{int}(disp), electronic interaction energy E_{int}(elec), and preparation energy E_{prep} in dependence of the adsorption height D_{ads} for **anc** (solid line) and **pht** (dashed line) obtained by model calculations where only the H atoms were left to freely optimize.

7 Projected Density of States of anc

In the projected density of states (PDOS) of **anc** adsorbed on the Cu(111), multiple peaks can be seen (Figure S6). In order to assign the molecular orbitals (MOs) of **anc** to these peaks, the partial charge density (corresponding to the local density of states) was determined at the respective peak energy (±0.05 eV) and compared with the gas phase MOs (each 1 Å above the molecular plane).
The peak at -1.30 eV can be clearly assigned to the HOMO of **anc** and the two peaks at 0.29 and 0.73 eV can be clearly assigned to the LUMO. Furthermore, the LUMO+2 can be assigned to the peak at 1.97 eV. The LUMO+1 might be visible at 1.27 eV with contributions from the LUMO and/or LUMO+2. The HOMO-2 and HOMO-1 might overlap at the peak at -3.77 eV as there are not states located at the central ring of **anc**. The remaining peaks do not show any characteristics of the MOs of **anc** and are therefore considered as artefacts.
This shows that the LUMO is quite close to the Fermi energy E_F. Therefore, a slight charge transfer into the LUMO as observed by the Hirshfeld charge analysis seems plausible. Still, this charge transfer is significantly lower than for chemisorbed molecules, where a hybridization between the LUMO and the surface states is assumed.

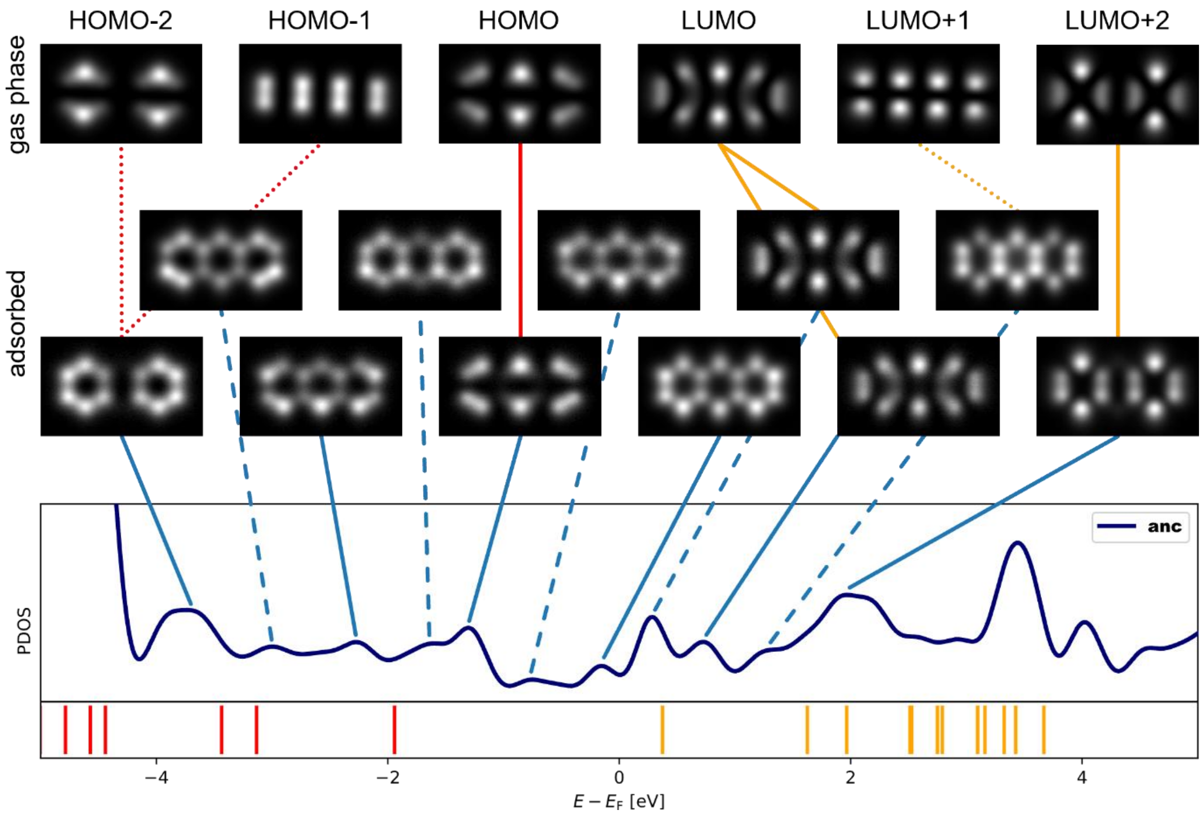

Figure S6. Projected density of states (PDOS) of **anc** on the Cu(111) surface (dark blue). Vertical lines below show the occupied (red) and virtual (orange) gas phase molecular orbital energies aligned to the assigned peaks in the PDOS. Local density of states (LDOS) above were used to assign the peaks in the PDOS to the molecular orbitals.

8 Top View on Optimized Adsorption Structures

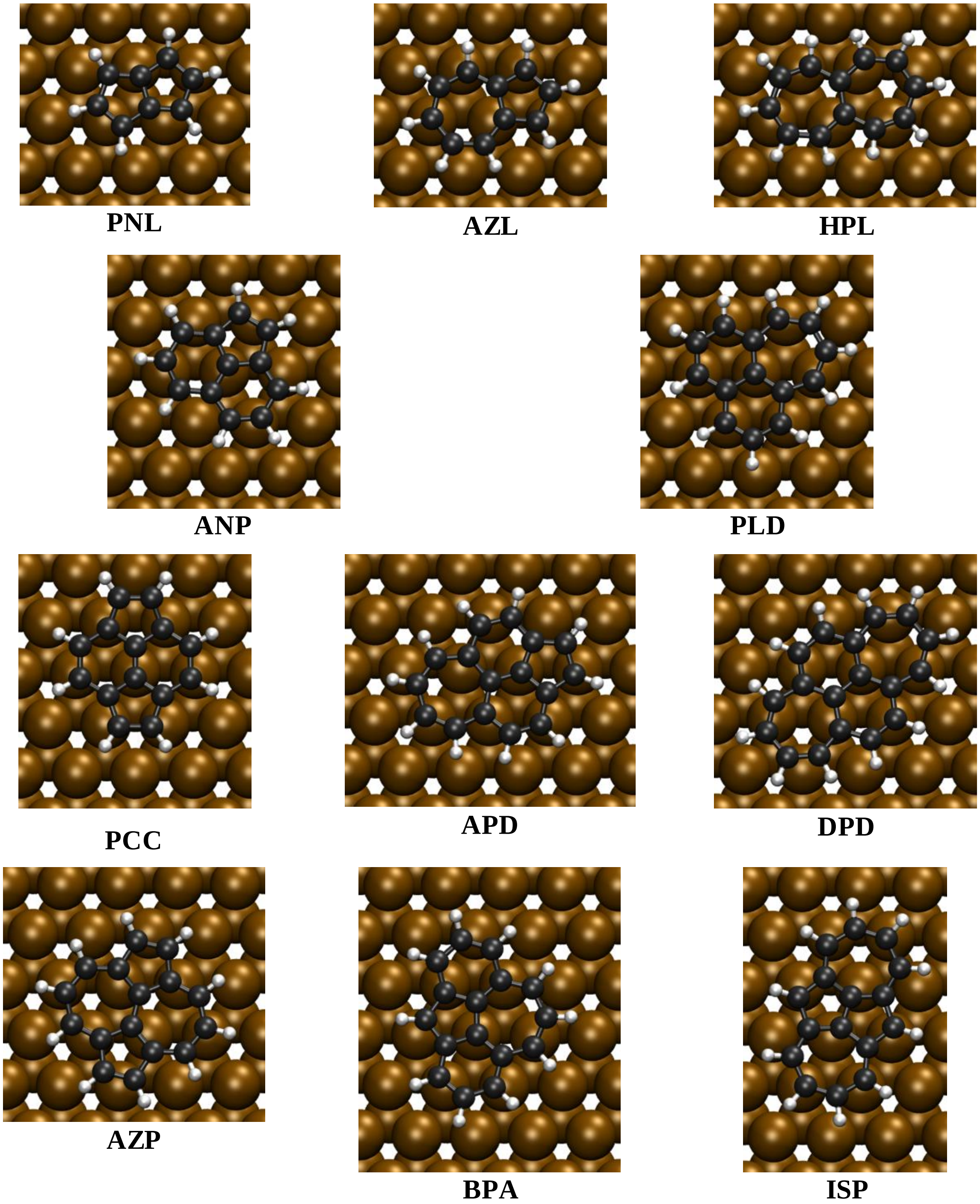

Figure S7. Top view on adsorbed non-alternant π-electron systems on Cu(111) surface, with the corresponding acronyms being defined in Scheme 1 of the manuscript. Note that only the top two layers of the Cu(111) slab are shown, so that the fcc hollow sites have no Cu atoms underneath, while the hcp hollow sites do.

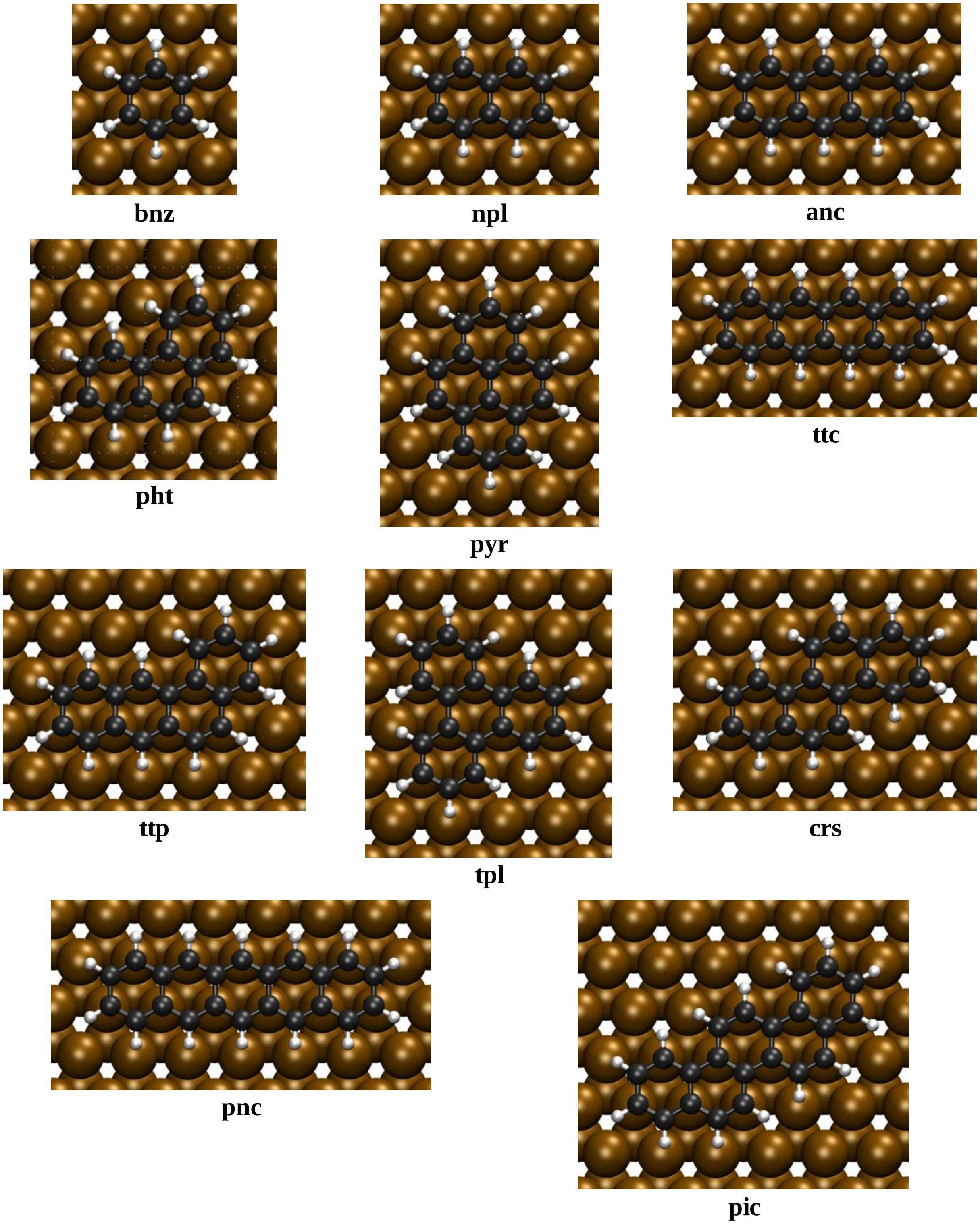

Figure S8. Top view on adsorbed alternant π-electron systems on Cu(111) surface, with the corresponding acronyms being defined in Scheme 1 of the manuscript. Note that only the top two layers of the Cu(111) slab are shown, so that the fcc hollow sites have no Cu atoms underneath, while the hcp hollow sites do.

9 Data used for Analysis

Table S1. Simple structural properties of the investigated π-electron systems.[a]

Name	#C	#H	#Atoms	#Rings
**PNL**	8	6	14	2
**AZL**	10	8	18	2
**HPL**	12	10	22	2
**ANP**	12	8	20	3
**PLD**	14	10	24	3
**PCC**	14	8	22	4
**APD**	16	10	26	4
**DPD**	18	12	30	4
**AZP**	16	10	26	4
**BPA**	16	10	26	4
**ISP**	16	10	26	4
**bnz**	6	6	12	1
**npl**	10	8	18	2
**anc**	14	10	24	3
**pht**	14	10	24	3
**pyr**	16	10	26	4
**ttc**	18	12	30	4
**ttp**	18	12	30	4
**tpl**	18	12	30	4
**crs**	18	12	30	4
**pnc**	22	14	36	5
**pic**	22	14	36	5

[a] Number of carbon atoms #C, number of hydrogen atoms #H, number of total atoms #Atoms, and number of rings #Rings.

Table S2. Energetic interface properties for the Cu(111)-organic interface.[a]

Name	E_{ads}	E_{prep}	E_{prep}(surf)	E_{prep}(mol)[b]	E_{int}	E_{int}(disp)[c]	E_{int}(elec)
**PNL**	-291.6	77.9	17.4	60.5 (78%)	-369.5	-144.6 (39%)	-225.0
**AZL**	-200.6	62.0	16.8	45.2 (73%)	-262.6	-183.5 (70%)	-79.1
**HPL**	-297.3	75.0	25.0	50.0 (67%)	-372.4	-218.3 (59%)	-154.1
**ANP**	-207.4	44.3	14.9	29.4 (66%)	-251.7	-210.1 (83%)	-41.6
**PLD**	-237.9	42.0	15.0	27.0 (64%)	-279.9	-250.5 (89%)	-29.4
**PCC**	-286.3	89.3	24.1	65.1 (73%)	-375.5	-233.2 (62%)	-142.3
**APD**	-244.7	43.1	17.6	25.5 (59%)	-287.8	-276.4 (96%)	-11.4
**DPD**	-319.8	60.6	20.6	40.0 (66%)	-380.4	-317.4 (83%)	-63.0
**AZP**	-261.2	63.1	19.4	43.7 (69%)	-324.3	-269.6 (83%)	-54.7
**BPA**	-344.6	64.9	20.2	44.8 (69%)	-409.5	-275.0 (67%)	-134.6
**ISP**	-284.8	74.3	27.4	46.9 (63%)	-359.1	-274.4 (76%)	-84.8
**bnz**	-104.3	4.8	2.7	2.1 (43%)	-109.1	-115.8 (106%)	6.7
**npl**	-154.4	4.7	2.9	1.8 (38%)	-159.1	-174.4 (110%)	15.3
**anc**	-211.1	13.0	6.3	6.6 (51%)	-224.1	-246.5 (110%)	22.4
**pht**	-204.4	4.8	3.1	1.7 (35%)	-209.2	-230.9 (110%)	21.7
**pyr**	-220.5	3.6	2.5	1.1 (31%)	-224.0	-247.1 (110%)	23.1
**ttc**	-275.7	48.4	16.1	32.3 (67%)	-324.1	-315.0 (97%)	-9.1
**ttp**	-258.3	7.1	4.0	3.0 (43%)	-265.3	-293.2 (111%)	27.9
**tpl**	-257.5	7.2	4.3	2.8 (40%)	-264.7	-294.7 (111%)	30.0
**crs**	-254.0	5.2	3.4	1.8 (34%)	-259.2	-288.4 (111%)	29.2
**pnc**	-347.2	52.4	17.4	35.0 (67%)	-399.6	-378.5 (95%)	-21.1
**pic**	-304.1	5.8	3.8	2.0 (35%)	-309.9	-344.6 (111%)	34.7

[a] Adsorption energy E_{ads}, total preparation energy E_{prep}, surface preparation energy E_{prep}(surf), molecular preparation energy E_{prep}(mol), total interaction energy E_{int}, dispersion interaction energy E_{int}(disp), and electronic interaction energy E_{int}(elec). All energies in kJ/mol.
[b] Percentage values give the contribution of E_{prep}(mol) relative to E_{prep}.
[c] Percentage values give the contribution of E_{int}(disp) relative to E_{int}.

Table S3. Geometric and electronic interface properties for the Cu(111)-organic interface.[a]

Name	D_{ads}	D_{ads}^{MAD}	θ_{bend}	Δq	$\Delta\Phi$
**PNL**	2.145	0.048	15.31	-0.248	-0.3046
**AZL**	2.247	0.045	11.88	-0.216	-0.3743
**HPL**	2.235	0.057	10.02	-0.331	-0.4014
**ANP**	2.362	0.088	9.69	-0.178	-0.3992
**PLD**	2.418	0.096	6.94	-0.199	-0.4458
**PCC**	2.250	0.087	13.68	-0.348	-0.4009
**APD**	2.394	0.043	8.20	-0.243	-0.4868
**DPD**	2.404	0.064	6.85	-0.354	-0.4829
**AZP**	2.303	0.068	10.64	-0.320	-0.4829
**BPA**	2.317	0.086	10.49	-0.362	-0.4469
**ISP**	2.243	0.095	12.97	-0.364	-0.5020
**bnz**	2.609	0.002	2.46	0.018	-0.2597
**npl**	2.698	0.010	1.78	0.011	-0.3519
**anc**	2.595	0.037	3.06	-0.062	-0.4507
**pht**	2.763	0.033	0.97	-0.004	-0.4073
**pyr**	2.834	0.015	0.31	0.001	-0.4191
**ttc**	2.431	0.098	5.89	-0.292	-0.5066
**ttp**	2.755	0.047	1.05	-0.035	-0.4883
**tpl**	2.746	0.021	0.68	-0.041	-0.4898
**crs**	2.791	0.022	0.55	-0.023	-0.5062
**pnc**	2.456	0.095	5.18	-0.386	-0.5458
**pic**	2.817	0.035	0.73	-0.036	-0.5442

[a] Adsorption height D_{ads} and molecular corrugation D_{ads}^{MAD}, each in Å. Bending angle θ_{bend} in °. Charge transfer Δq in e (negative value corresponds to electron excess on molecule). Change in work function $\Delta\Phi$ in eV.
